# Bioactivity Focus of α-Cyano-4-hydroxycinnamic acid (CHCA) Leads to Effective Multifunctional Aldose Reductase Inhibitors

**DOI:** 10.1038/srep24942

**Published:** 2016-04-25

**Authors:** Laitao Zhang, Yi-Fang Li, Sheng Yuan, Shijie Zhang, Huanhuan Zheng, Jie Liu, Pinghua Sun, Yijun Gu, Hiroshi Kurihara, Rong-Rong He, Heru Chen

**Affiliations:** 1 Institute of Traditional Chinese Medicine and Natural Product, College of Pharmacy, Jinan University, Guangzhou 510632, P. R. China; 2 Guangdong Province Key Laboratory of Pharmacodynamic Constituents of TCM and New Drugs Research, Guangzhou 510632, P. R. China; 3 Institute of Clinical Pharmacology, Guangzhou University of Chinese Medicine, Guangzhou 510006, P. R. China; 4 National Center for Protein Science Shanghai, Shanghai 201210, P. R. China; 5 State key Laboratory of Drug Research, Shanghai Institute of Materia Medica, Chinese Academy of Sciences, Shanghai 201203, P. R. China

## Abstract

Bioactivity focus on α-cyano-4-hydroxycinnamic acid (CHCA) scaffold results in a small library of novel multifunctional aldose reductase (ALR2) inhibitors. All the entities displayed good to excellent inhibition with IC_50_ 72–405 nM. (*R*,*E*)-*N*-(3-(2-acetamido-3-(benzyloxy)propanamido)propyl)-2-cyano-3-(4-hydroxy phenyl)acrylamide (**5f**) was confirmed as the most active inhibitor (IC_50_ 72.7 ± 1.6 nM), and the best antioxidant. **5f** bound to ALR2 with new mode without affecting the aldehyde reductase (ALR1) activity, implicating high selectivity to ALR2. **5f** was demonstrated as both an effective ALR2 inhibitor (ARI) and antioxidant in a chick embryo model of hyperglycemia. It attenuated hyperglycemia-induced incidence of neural tube defects (NTD) and death rate, and significantly improved the body weight and morphology of the embryos. **5f** restored the expression of paired box type 3 transcription factor (Pax3), and reduced the hyperglycemia-induced increase of ALR2 activity, sorbitol accumulation, and the generation of ROS and MDA to normal levels. All the evidences support that **5f** may be a potential agent to treat diabetic complications.

Diabetes mellitus (DM) is a chronic disease characterized by glucose metabolic dysfunction and hyperglycemia. Its incidence is now growing at an almost epidemic rate all over the world. In diabetic patients, aldose reductase (ALR2) is activated to turn glucose into sorbitol, which is subsequently converted to fructose by sorbitol dehydrogenase. This so called polyol pathway results in overproduction of the organic osmolyte sorbitol and the depletion of NADPH cell stores, in which the later process may eventually increase the susceptibility of cells to damage by reactive oxygen species (ROS). Formation of fructose (which is a reactive glycation agent) in the polyol pathway contributes to the glycation process. As a consequence, diabetic complications such as neuropathy, retinopathy, cataracts, nephropathy, and cardiovascular diseases will probably be induced^1^,^2^,^3^,^4^,^5^,^6^,^7^,^8^,^9^,^10^. This is why ALR2 has long been regarded as an attractive therapeutic target for designing drugs to counteract the development of long-term diabetic complications.

In another aspect, ROS is closely related to many diseases. As we know, endogenous ROS is produced during cellular metabolism and functional activities and has important roles in cell signaling, apoptosis, gene expression, and ion transportation^11^. However, pathological ROS gives rise to oxidative stress leading to the oxidative degradation of vital biomolecules such as lipids, proteins, and DNA. Consequently this contributes to the pathogenesis of inflammatory disease, cardiovascular disease, cancer, diabetes, cataracts, Alzheimer’s disease, and aging^12^,^13^,^14^,^15^. This is one of the reasons why antioxidants may prevent cellular damage from oxidative stress and also lower the risk of chronic diseases.

So far, a great variety of aldose reductase inhibitors (ARIs) have been identified and many of them have been evaluated in preclinical and clinical trials^16^,^17^,^18^,^19^,^20^. Although fidarestat is currently regarded as one of the most promising drug candidate among ARIs^2^,^9^,^21^, many of them were unfortunately hampered by pharmacokinetic drawbacks, low *in vivo* efficacy, or adverse side effects^22^,^23^. At present, only epalrestat (Supplementary Fig. S1) is authorized in a few Asian countries (such as Japan, China and India) as a commercially available ARI for the treatment of diabetic neuropathy^24^. No doubt, more efforts should be made to develop more effective ARIs with improved therapeutic potential.

From a viewpoint of chemical structure, most of the reported ARIs are compact rigid compounds, such as sorbinil, imirestat, tolrestat, zopolrestat, and zenarestat. It is noteworthy that some side effects including hypersensitivity reactions and liver toxicity have been confirmed related to the fused hydantoin ring in the structure^25^,^26^,^27^.

We notice the strategy that using natural lead molecules as a design base may give a better chance to discover lead compounds displaying desirable biological and pharmacological properties^28^,^29^,^30^,^31^, which resulted in our focus on several simple molecules from plant resources (Supplementary Fig. S1) that display AR inhibition activities^31^,^32^,^33^,^34^. They share a common *p*-coumaric acid scaffold, which has proven to be as a good AR inhibitor (IC_50_ 0.14 μM)^33^. Unfortunately *p*-coumaric acid is considered reactive, membrane-active, non-specific, or even highly promiscuous^35^,^36^,^37^. However, if we limit or focus their biological activities through structural modification, this molecule could be used as the basic structure to design potential ARIs with functions against multiple targets. We call this strategy as bioactivity focus.

A previous docking study using the crystal structure of the human aldose reductase complexed with NADP^+^ and IDD-type inhibitor (PDB: 2IKI)^38^ was performed. We found that the introduction of a cyano group at the α-position of *p*-coumaric acid will enhance the binding affinity with two more hydrogen bonds: –CN/S214 and –C = O/S214 (Fig. 1A). Therefore, α-cyano-4-hydroxycinnamic acid (CHCA) is more suitable as the basic scaffold in designing ARIs. Since ALR2 has three distinct binding sites (the substrate binding site, the nucleotide cofactor (NADP^+^) fold, and the inhibitor binding site)^39^ and most of the reported ARIs bind to the common inhibitor binding site^40^, we made a bold vision that if an appropriate structure is incorporated into CHCA, the new entity may occupy both the cofactor and inhibitor binding sites resulting in enhancement of binding affinity and selectivity (Fig. 1B).

Therefore, in the present study, CHCA was applied as the building scaffold in a series of novel flexible ARIs. The chemistry and biology of these new compounds will be described.

## Results

### Chemistry

**Figure 2:**
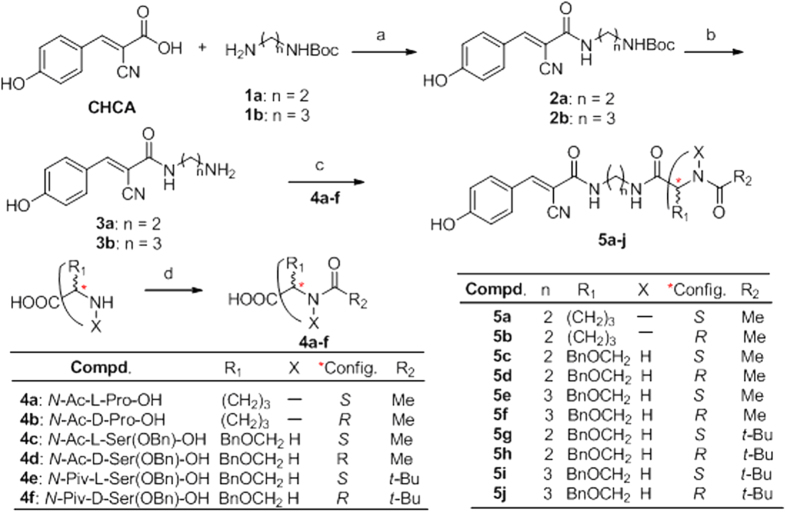
Design and synthesis of novel flexible ARIs. Reaction conditions: a. EDCI·HCl/HOBt, DCM/DMF (10:1, *V/V*), 62–63%; b. 4 M HCl/methanol, >98%; c. EDCI·HCl/HOBt, DCM/DMF (10:1, *V/V*), 65–76%; d. Ac_2_O or Piv_2_O, reflux, 83–95%.

The novel flexible ARIs consist of a CHCA moiety, a spacer, and a modified amino acid part (MAA). Their syntheses are outlined in Fig. 2. Without the protection of the 4-hydroxyl group, CHCA reacted directly with monoamino protected diamines with 1-ethyl-3-(3-dimethylaminopropyl)carbodiimide hydro- chloride (EDC·HCl) as a coupling reagent, which led to **2a–b**. The yields were 62–63%. Deprotection of **2a–b** was carried out smoothly by treatment with 4 mol/L HCl in methanol solution. This was a clean and almost quantitative reaction affording **3a–b** in more than 98% yield after removal of excess solvent. **4a**,**b** are commercially available. **4c**,**d** were obtained by the reaction of acetic anhydride with *O*-benzyl-L-serine, and *O*-benzyl-D-serine, respectively. Both yields were 94.5%. Preparation of **4e**,**f** was similar to that of **4c–d**, in which acetic anhydride was replaced with pivalic anhydride. The yields were decreased, which were both at 83.4%. The final compounds **5a–j** were prepared by the reaction of **3a–b** with **4a–f** under the coupling of EDC·HCl, respectively. The yields were 61–73%.

### ALR2 inhibition and structure-activity relationship (SAR)

Compounds **5a–j** were evaluated for their potential to inhibit the *in vitro* reduction of D,L-glyceraldehyde by human ALR2 purified from the culture medium of a baculovirus-insect cell expression system, using epalrestat as a positive reference (Table 1). All the tested compounds (**5a–j**) displayed inhibition activities with IC_50_ values of 72–405 nM. **5f** was confirmed as the most active inhibitor with IC_50_ 72.7 ± 1.6 nM, which was almost equal to that of the reference (IC_50_ 61.3 ± 1.3 nM).

The current flexible ARIs consist of CHCA moiety, spacer, and a MAA. As indicated in Table 2 **lane 2**, it is noteworthy that an appropriate carbon chain length is very important for the ALR2 inhibition. For example, the inhibition activity of **5f** was about 5 times higher than that of **5d**, while the structural difference between them is only one more methylene in the spacer. Comparing **5e** with **5i**, and **5f** with **5j**, respectively, it seemed that bulky groups like *tert*-butyl decrease the binding affinity. However, in the case of a shorter spacer (**5c** with **5g**, **5d** with **5h**, n = 2), the existence of a *tert*-butyl unexpectedly increases the binding affinity of the inhibitor to the enzyme. Although **5a**,**b** also had shorter carbon chains, both compounds displayed stronger activity than **CHCA** itself. It is likely that there is an exquisite match between inhibitor and enzyme. Fine adjustment of the spacer and MAA has great impact on the binding affinity. In order to explore SAR thoroughly, more compounds need to be synthesized. And it is on the progress in our group now.

So far from the present data, it seemed that the chirality in the MAA had only a slight influence on the inhibition activity, indicating that the binding pocket for this hydrophobic part is not sensitive to chirality.

In an attempt to gain insight into the structural basis of the binding interaction between the substrate and AR, we performed molecular docking studies of all the compounds. When only the inhibitor binding pocket was applied for the docking evaluation, it was found that all the ligands projected toward the solvent area through the groove above the nicotinamide binding pocket with a poor docking score, which meaned poor binding between the substract and enzyme. This was absolutely not in agreement with the sub-micromolar levels of IC_50_ values as shown in Table 1 **lane 2**. Therefore, we speculated that some ligands might bind to multiple pockets of ALR2, which led to the enhancement of the binding affinity.

Based on this assumption, the docking studies were carried out. The binding affinity and docking scores were shown in Table 1 **lane 3**–**4**. It was found that both orders were quite coincident with the order of inhibition activities. By inspecting the conformation of docked ligands, we discovered that the binding mode of the ten flexible ARIs could be categorized into two types.

**Figure 3:**
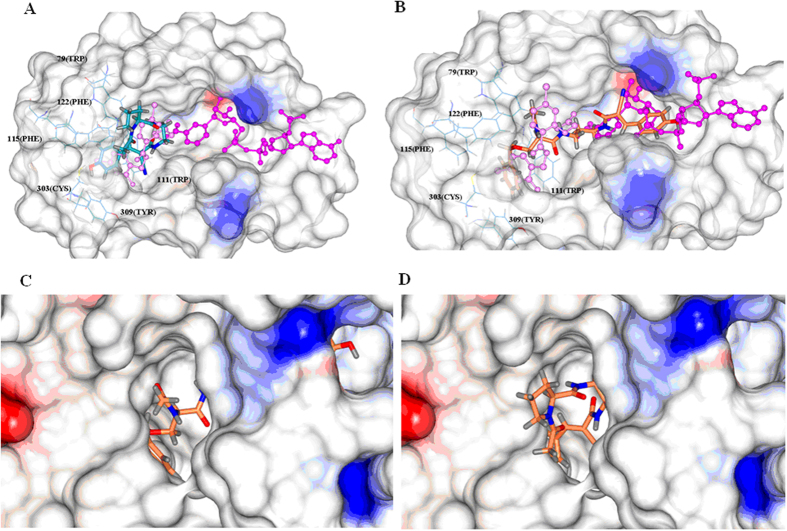
The two categories of ligand binding modes. (**A**) **5a** represents the first binding mode, occupying only the inhibitor binding pocket. Pink: IDD inhibitor; Magenta: NADP+; Dark cyan: **5a**. (**B**) **5f** represents the alternative binding mode, occupying both the inhibitor and NADP^+^ binding pockets. Pink: IDD inhibitor; Magenta: NADP+; Coral: **5f**. (**C**) The surface representation of electrostatic potential of aldose reductase complexed with **5a**. (**D**) The surface representation of electrostatic potential of aldose reductase complexed with **5f**.

Compounds **5a, 5b, 5d, 5h** and **5g**, with shorter spacer and more cmpacted structures, adopted the first category of binding mode. As schematically represented by **5a** in Fig. 3A,C, the aromatic *p*-hydroxybenzyl group of **5a** occupies the hydrophobic pocket of ALR2 enclosed by the side chains of L300, Y309, C303, F115, F122 and W111, forming one H-bond with T113 OG1 with a distance of 3.2 Å; and the remaining part of the molecule occupied a groove flanked by the side chains of Y48, W20, and V47 right above the nicotinamide binding pocket.

The second binding mode was adopted by **5c, 5e, 5f, 5i** and **5j**, which were structurally characterized by longer spacer and more flexibility. Taking the binding mode of the most potent compound **5f** as an example, the mode was detailed in Fig. 3B,D. Compared to the first category of ligands, it was obvious that **5f** adopted a very different conformation. The benzyl group occupied the same hydrophobic pocket enclosed by the side chains of L300, Y309, C303, F115, F122 and W111 forming one H-bond between the hydroxyl group of the ligand and T113 OG1 with the distance of 3.0 Å. However, the *p*-hydroxybenzyl group projected toward the nicotinamide binding pocket, being buried by the side chains of W20, I210, S210, V297, D43.

### Antioxidative capacity

With the CHCA scaffold, we hypothesized that **5a–j** should have antioxidant activity. Therefore, a radical absorbance capacity assay (ORAC_FL_)^41^ was employed to assess their capacities. This method is based on the inhibition of peroxyl-radical-induced oxidation initiated by thermal decomposition of azo-compounds, in this case 2,2′-azo-bis (2-amidinopropane) dihydrochloride (AAPH). Trolox (6-hydroxy-2,5,7,8-tetramethylchroman-2-carboxylic acid) was used as a standard reference. Data is expressed as micromoles of Trolox equivalents (TE) per microliter of sample (μmol TE/ml or U/ml). As indicated in Table 1 **lane 5**, **5f**, the most potent ARI, was also the most active antioxidant when tested at a concentration of 3.125 μM. It displayed an ORAC value of 65.7 U/ml which was quite close to that of mangiferin, and was only slightly less active than that of Trolox. Quite interestingly, the antioxidative capacity was positively correlated with the ALR2 inhibition activity. That is, candidate with higher ORAC value showed greater inhibition.

### Stability and toxicity

Predicted from its structure, the stability of **5f** will be good and the toxicity will be low. By using quantitative RP-HPLC analysis after treatment of **5f** in Dulbecco’s modified Eagle’s medium (DMEM) containing 5% fetal bovine serum (FBS) at 37 °C over time, we found that **5f** was quite stable under these conditions (Supplementary A of Fig. S2). After 12 h incubation, 86% of **5f** remained intact.

In another aspect, the toxicity was evaluated by the growth inhibition of **5f** against human embryonic kidney 293 (HEK293) cells. It was shown that **5f** was nontoxic to HEK293 cells even at high concentration of 500 μM (Supplementary B of Fig. S2). The inhibition rate was only 19.2%. Evidently, epalrestat displayed stronger toxicity at the same concentration and the inhibition rate was 36.4% determined at 500 μM (Supplementary B of Fig. S2).

### 5f prevented the damage of embryos exposed to high glucose

**Figure 4:**
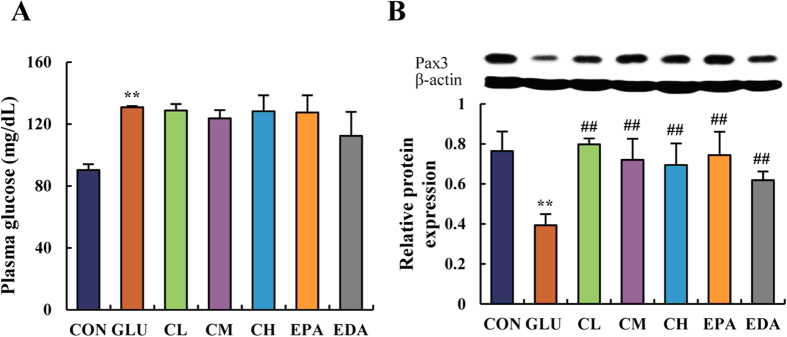
5f restored neural tube development and marker expression in chick embryos on EDD 5. Glucose concentration (**A**) and Pax3 protein expression (**B**) were determined in chick embryos. The plasma glucose concentration was measured using a glucose oxidase-coupled spectrophotometric assay kit. Proteins were detected using the monoclonal antibody anti-Pax3 diluted 1:1000 (DSHB, USA) and visualized using anti-mouse IgG conjugated with horseradish peroxidase (HRP) and Pierce ECL Western Blotting Substrate (Thermo Fisher Scientific, USA) as the substrate of HRP. The abbreviations “CON”, “GLU”, “CL”, “CM”, “CH”, “EPA”, “EDA” mean “controlled”, “glucose treated”, “low concentration of **5f** treated”, “mild concentration of **5f** treated”, “high concentration of **5f** treated”, “epalrestat treated”, “edaravone treated” groups, respectively. Values were expressed as mean ± SD in each group (n = 10). *P < 0.05, **P < 0.01 *vs*. control. ^#^*P* < 0.05, ^##^*P* < 0.01 *vs*. glucose.

In order to evaluate further the potential of **5f** as an agent to treat diseases caused by high glucose inside the system, a chick embryo model of treatment with high glucose was set up. As shown in Fig. 4A, after glucose (0.4 mmol/egg) injection on the first day of embryo development (EDD 1), the concentration of plasma glucose was significantly increased. In the glucose-treated EDD 5 embryos, the death rate and occurrence of neural tube defects (NTD) were significantly increased (40% and 58.3%, respectively); while the body weight decreased (Table 2). High glucose exposure also caused morphology changes in the whole mount embryo, incomplete closure of the neural tube (Supplementary B1 and B2 of Fig. S3), and the decrease of paired box type 3 transcription factor (Pax3) expression (Fig. 4B), which is an evolutionary conserved transcription factor that belongs to the paired box family playing a pivotal role during neural tube development^42^.

Excitingly, **5f** did lower down the hyperglycemia-induced death and NTD rates, and recovered the body weights of EDD 5 embryos in a dose-dependent manner (Table 2). At the same dose (1 μM), **5f** displayed slightly better effects than epalrestat (EPA), especially on NTD. Edaravone (EDA), another positive reference, is a nootropic and neuroprotective agent used for the purpose of aiding neurological recovery following acute brain ischemia and subsequent cerebral infarction^43^. It displayed slightly better effects than **5f**. Moreover, **5f** recovered the morphology of the embryo and prevented abnormal closure of the neural tube (Supplementary C1 and C2 of Fig. S3). It also restored Pax3 expression (Fig. 4B). However, whether **5f** attenuates NTD through the adjustment of Pax3 requires further investigation. As a comparison, both EPA and EDA displayed similar effects. Of particular note, **5f** did not lower the sustained high level of plasma glucose (Fig. 4A). This implied that **5f** could not be applied as a hypoglycemic agent. Neither could EPA nor EDA.

### 5f reduced AR activity and sorbitol content in glucose-treated embryo

**Figure 5:**
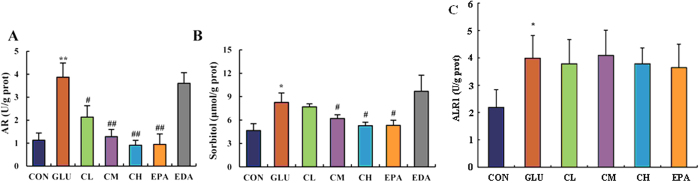
Effects of **5f** on ALR2 activity, sorbitol content, and aldehyde reductase (ALR1) in high-glucose-treated embryos on EDD 5. ALR2 activity (**A**), sorbitol content (**B**), and ALR1 activity (**C**) of embryo on EDD 5 were detected. The abbreviations “CON”, “GLU”, “CL”, “CM”, “CH”, “EPA”, “EDA” means the same as depicted in Fig. 4. Values were expressed as mean ± SD in each group (n = 10). *P < 0.05, **P < 0.01 *vs*. control, ^#^*P* < 0.05, ^##^*P* < 0.01 *vs*. glucose.

In the current glucose-treated embryo model, it was clearly demonstrated that the activity of ALR2 was increased from 1.2 U/g prot to 3.9 U/g prot, and the concentration of sorbitol from 3.5 μmol/ g prot to 8.4 μmol/g prot (Fig. 5A,B). As expected, treatment of **5f** decreased ALR2 activity in a dose-dependent manner. When treated at the dose of 1 μM, **5f** adjusted ALR2 activity to a level that is close to that of the control (Fig. 5A). As a result, sorbitol content was decreased significantly (Fig. 5B). EPA, which is a clinical ARI, had the similar effects to **5f**. However, EDA, which is a clinical radical scavenger, was shown no effect on the inhibition of ALR2.

### 5f did not affect aldehyde reductase (ALR1) level in glucose-treated embryo

As shown in Fig. 5C, a high concentration of glucose significantly elevated ALR1 level in chicken embryo (*P* < 0.01). Compared with the model group, there was no obvious change in **5f** and epalrestat treatment groups. This evidence indicated that **5f** was high selective to ALR2.

### 5f reduced the hyperglycemia-induced oxidative stress in embryos

**Figure 6:**
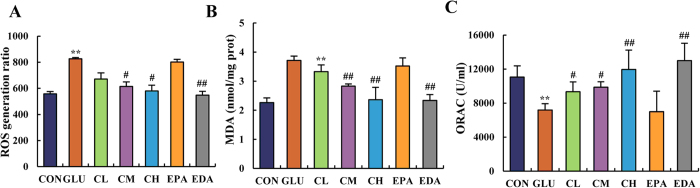
5f ameliorated the oxidative stress state induced by high glucose in chick embryos on EDD 5. ROS generation ratio (**A**), MDA level (**B**) and ORAC level (**C**) were measured in chick embryo on EDD 5. The abbreviations “CON”, “GLU”, “CL”, “CM”, “CH”, “EPA”, “EDA” means the same as depicted in Fig. 4. Values were expressed as mean ± SD in each group (n = 10). *P < 0.05, **P < 0.01 *vs*. control, ^#^*P* < 0.05, ^##^*P* < 0.01 *vs*. glucose.

It has been reported that hyperglycemia increased the production of ROS. Blocking the generation of ROS could at least partially prevent the possible damage of hyperglycemia^44^. No doubt, oxidative stress will be induced in glucose treated embryos. This conclusion was confirmed by the determination of ROS and malondialdehyde (MDA) levels, and the anti-oxidation ability in chick embryos on EDD 5. As indicated in Fig. 6A,B, high glucose treatment significantly increased the generation of ROS and MDA. However, treatment of **5f** decreased both levels of ROS and MDA in a dose-dependent manner. When treated at the dose of 1 μM, **5f** attenuated both ROS and MDA levels to a normal state, which was comparable to that of the control group. As an antioxidant in clinic, EDA displayed the same effect at the dose of 0.1 nM; while EPA showed no effect.

In addition, the ORAC level is indicative of the anti-oxidative capacity. As shown in Fig. 6C, the ORAC level in a glucose-treated chick embryo was lower than that in controlled group. As expected, **5f**, as well as EDA, recovered the anti-oxidative capacity of embryo in dose-dependent manner, while EPA showed no effect.

## Discussion

In the present investigations, we have verified that CHCA is a good scaffold for the design of novel ARIs. With this scaffold in the new entity, it guarantees a certain extent of ALR2 inhibition activity and antioxidant capacity. The incorporation of a suitable carbon chain and modified amino acid moiety resulted in good ARI candidate. So far, (*R*,*E*)-*N*-(3-(2-acetamido-3-(benzyloxy)propanamido)propyl)-2-cyano-3-(4- hydroxy-phenyl)acrylamide (**5f**) has been confirmed as the most potent ARI. It is comparable to epalrestat, which is a clinically applied ARI agent.

Out of ordinary, **5f** has neither compact rigid structure nor free carboxylic group inside the molecule. However, it was shown with high and selective binding interaction with ALR2. We suggest that **5f** may have a special binding mode, in which both inhibitor and NADP^+^ binding sites of ALR2 are occupied. With this mode it will no doubt enhance the binding affinity and selectivity of **5f** to ALR2. Of course, whether this multiple binding mode is true or not requires further investigations. As a matter of fact, co-crystallization of ALR2 with **5f** is now on the progress by our research group.

Because of the sequence homology and the nonspecificity of inhibition between ALR2 and ALR1, it has been suggested that the inhibitor binding sites of both enzymes are structurally similar^45^. Therefore, many ARIs are reported nonspecific. That is, they can inhibit either ALR2 or ALR1. Fortunately, as well as EPA, **5f** did not affect the hyperglycemia-elevated ALR1 level. This data suggested the high selective inhibition of **5f** to ALR2. At this point, we conclude that **5f**, which is a candidate compound modified based on CHCA, has been confirmed not a promiscuous molecular with binding selectivity between ALR2 and ALR1.

Pax3 is a gene that belongs to the paired box family of transcription factors expressed in early embryonic phases, which plays key roles in neural tube ontogenesis^46^. Mutation of Pax3 can cause multiple defects in neural crest and neural tube tissues. In mouse embryos collected from hyperglycemic dames whose hyperglycemia was induced with either streptozotocin (STZ: 2-deoxy-2-(3 -methyl-3-nitrosoureido)-d-glucopyranose) or phlorizin-treatments, a high incidence of congenital neural tube defects and reduced levels of Pax-3 mRNA have been reported; and reduced expression of Pax-3 was associated with apoptosis, as measured by TUNEL-labeled DNA fragments, within the neuroepithelial of the mouse neural tube^47^. In the present study, the expression levels of the Pax3 protein were found diminished in high glucose-treated embryos. Quite interestingly, supplementation with **5f** as well as EPA and EDA could significantly restore the expression levels of Pax3 in hyperglycemic chick embryos. As a result, treatment with **5f**, EPA, and EDA, respectively, were able to significantly ameliorate the hyperglycemia-induced NTD rate. This evidence supports that **5f** may potentially act as an agent to treat hyperglycemia-induced neurogenic diseases in clinic as both EPA and EDA do.

Investigations showed that hyperglycemia increase the activity of the polyol pathway leading to the excess generation of sorbitol, which poorly diffuses across the cell membrane resulting in cell damage^48^. Substantially, our results indicated that treatment with **5f**, as well as EPA, could adjust the activity of ALR2 and the concentration of sorbitol in glucose-treated embryo to levels that were closed to normal state. This evidence supports that **5f** is a potential agent to treat diseases caused by the disorder of ALR2.

We demonstrated here that exogenous glucose can induce oxidative stress and promote decreased viability in chick embryos. As mentioned above, exogenous glucose also induced the accumulation of sorbitol in diabetic chick enbryos, which might in turn lead to oxidative stress^49^. ROS and MDA are two indicators of oxidative stress. We found that under the above-mentioned conditions, the levels of both indicators increased. ROS is thought to primarily exert its deleterious effects by damaging virtually all classes of biomolecules (including DNA, proteins and lipids) leading to cell death^50^. Therefore, ROS-induced oxidative stress may lead to many diseases including diabetes and gestational diabetes mellitus (GDM)^51^. As expected, **5f** supplementation dose-dependently abrogated the high-glucose-associated alterations in oxidative stress parameters of the embryos. The effects were comparable to those of EDA, which is a radical scavenger used clinically. This data, together with the evidence as an excellent ARI, supported that **5f** may be applied as an effective agent to treat diabetic complications.

In one word, by applying bioactivity focus strategy, CHCA was modified into an effective and selective anti-diabetic complications agent (**5f**). Out of the ordinary inhibtion binding mode, the new flexible candidate **5f** may probably bind to the nicotinamide binding pocket and a nearby hydrophobic pocket enclosed by side chains of W111, F122 F115, L300, C303, P310 in ALR2. This new binding mode makes **5f** an excellent ARI.

**5f** was confirmed a stable nontoxic compound with good antioxidant capacity, indicated it an effective multifunctional ARI. When evaluated in hyperglycemia chick embryos model, it attenuated the incidence of NTD and death rate in glucose-treated embryos, and improved significantly the body weight and morphology of embryos. **5f** restored the protein expression level of Pax3. It adjusted the hyperglycemia-induced increases of ALR2 activity, sorbitol accumulation, ROS and MDA generation to normal levels. However, **5f** did not affect hyperglycemia-elevated ALR1 activity, implicating the high selective inhibition to ALR2. All the evidences support that **5f** may act as a potential agent to treat diabetic complications, where epalrestat and edaravone are employed currently.

## Materials and Methods

### Research governance

All animal care and experimental procedures were approved by the Laboratory Animal Ethics Committee of Jinan University (20141112017), and were in accordance with the National Institute of Health’s Guide for the Care and Use of Laboratory Animals (7th edition, USA).

### General Methods for Chemistry

All reagents and solvents were used as purchased from commercial sources or indicated otherwise. Flash chromatography was performed using silica gel (300 mesh). All reactions were monitored by TLC, using silica gel plates with the fluorescence F_254_ displayed by UV light visualization. ^1^H NMR and ^13^C NMR spectra were recorded on a Bruker AV-400 spectrometer or Bruker AV-300. Coupling constants (*J*) are expressed in hertz (Hz). Chemical shifts (δ) of NMR are reported in parts per million (ppm) units relative to an internal control (TMS). Low resolution ESI-MS data were recorded on a Finnigan LCQ Advantage MAX mass spectrometer and high resolution ESI-MS data on an Applied Biosystems Q-STAR Elite ESI-LC-MS/MS mass spectrometer. The purity of the compounds was determined by reverse phase high performance liquid chromatography (HPLC) analysis to be >95%. HPLC was performed on either LC-100 liquid chromatograph equipped with a tunable LC-100 UV detector (Shanghai Wufeng Inc., China) or Agilent 1200 series liquid chromatograph equipped with an Agilent 1200 Series UV detector (Agilent Technologies, USA). The columns used were Cosmosil 5C18 (Nacalai Tesque Inc., Japan) for general purification. A flow rate of 1.0 mL/min was used with mobile phase of MeOH in H_2_O with 0.1% modifier (ammonia or trifluoroacetate, *v/v*).

### (*E*)-*tert*-butyl (2-(2-cyano-3-(4-hydroxyphenyl)acrylamido)ethyl)carbamate (2a)

To a 50-ml round flask, CHCA (946.0 mg, 5.0 mmol), 1-hydroxybenzotriazole (HOBt) (810.8 mg, 6.0 mmol), and EDC·HCl (1.15 g, 6.0 mmol) were dissolved in 20 ml of DCM/DMF (10: 1, *V/V*) at 0 °C. The mixture was stirred for 10 min. Afterwards, *N*-*tert*-butoxylcarbonyl ethyldiamine (0.9 ml, 5.5 mmol) and diisopropylethylamine (DIPEA) (2.3 ml, 13.2 mmol) were added via syringe. The reaction continued for 30 min at 0 °C. Then the reaction mixture was stirred at room temperature for 24 h. The organic solvent was then removed through rotatory evaporation under vacuum. The resulting residue was purified by column chromatography over silica gel using EtOAc/petroleum (1:3, *V/V*), to afford pure compound **2a** as a slightly yellow solid (1.04 g, yield: 62.3%). ^1^H NMR (300 MHz, CD_3_OD) δ 7.96 (s, 1H), 7.79 (d, *J* = 9.0 Hz, 2H), 6.62 (d, *J* = 9.0 Hz, 2H), 3.41 (t, *J* = 6.0 Hz, 2H), 3.24 (t, *J* = 6.0 Hz, 2H), 1.45 (s, 9H); ^13^C NMR (75 MHz, CD_3_OD) δ 163.1, 162.2, 157.4, 151.4, 133.0, 123.4, 116.5, 115.8, 100.2, 78.8, 40.5, 39.3, 27.3; ESI-MS (*m/z*): calc. for C_17_H_21_N_3_O_4_+Na [M+Na]^+^ 354.1, expr. 354.1.

### (*E*)-*tert*-butyl (2-(2-cyano-3-(4-hydroxyphenyl)acrylamido)ethyl)carbamate (2b)

Using a similar procedure as described in **2a**, compound **2b** was obtained as a slight yellow solid (1.07 g, 62.1%). ^1^H NMR (300 MHz, CD_3_OD) δ 8.09 (s, 1H), 7.92 (dd, *J* = 9.0 Hz, 2H), 6.93 (dd, *J* = 9.0 Hz, 2H), 3.38 (t, *J* = 6.0 Hz, 2H), 3.12 (t, *J* = 6.0 Hz, 2H), 1.74 (m, 2H), 1.45 (s, 9H); ^13^C NMR (75 MHz, CD_3_OD) δ 162.8, 162.2, 157.2, 151.4, 133.0, 123.4, 116.5, 115.8, 100.2, 78.6, 37.4, 37.3, 29.4, 27.4; ESI-MS (*m/z*): calc. for C_18_H_23_N_3_O_4_+Na [M+Na]^+^ 368.2, expr. 368.2.

### (*E*)-*N*-(aminoethyl)-2-cyano-3-(4-hydroxyphenyl)acrylamide (3a)

Compound **2a** (0.993 g, 3.0 mmol) was dissolved in methanol (15 ml). Then an HCl solution with a concentration 4 mol/L (7.5 ml) was added dropwise. The reaction mixture was stirred at room temperature for 4 h. Excess reagent was removed through rotatory evaporation under vacuum. The resulted residue was pure enough to be used directly without further purification. This slight yellow solid was compound **3a** (0.68 g, 98.0%). ESI-MS (*m/z*): calc. for C_12_H_13_N_3_O_2_+H [M+H]^+^ 232.1, expr. 232.1.

### (*E*)-*N*-(aminopropyl)-2-cyano-3-(4-hydroxyphenyl)acrylamide (3b)

Using a similar procedure as described in **3a**, compound **3b** was obtained as a slightly yellow solid (0.728 g, 99.0%). ESI-MS (*m/z*): calc. for C_13_H_15_N_3_O_2_+H [M+H]^+^ 246.1, expr. 246.2.

### (*S*)-2-acetamido-3-(benzyloxy)propanoic acid (4c)

*O*-benzyl-L-serine (196.0 mg, 1.0 mmol) was dissolved in methanol (15 ml), following the addition of acetic anhydride (0.3 ml, 3.0 mmol) via syringe under stirring. The reaction mixture was refluxed at 70 °C for 7 h. After removal of the solvent through rotary evaporation, the resulting residue was purified by RP-HPLC (eluant: methanol/0.05% TFA in H2O = 1:1, *V/V*), leading to the colorless oil **4c** (224.2 mg, 94.5%). ^1^H NMR (300 MHz, CD_3_OD) δ 7.33 (m, 5H), 4.70 (t, *J* = 6.0 Hz, 1H), 4.52 (s, 2H), 3.87 (dd, *J* = 3.0 Hz, 2H), 2.02 (s, 3H); ^13^C NMR (75 MHz, CD_3_OD) δ 172.0, 170.7, 137.8, 128.1, 127.5, 127.5, 72.8, 69.4, 52.8, 21.2; ESI-MS (*m/z*): calc. for C_12_H_15_NO_4_+Na [M+Na]^+^ 260.1, expr. 260.2.

### (*R*)-2-acetamido-3-(benzyloxy)propanoic acid (4d)

Using a similar procedure as described in **4c** with *O*-benzyl-D-serine as a starting material, compound **4d** was obtained as a colorless oil (222.5 mg, 93.8%). ^1^H NMR (300 MHz, CD_3_OD) δ 7.32 (m, 5H), 4.68 (t, *J* = 6.0 Hz), 4.52 (s, 2H), 3.73 (dd, *J* = 3.0 Hz, 2H), 2.01 (s, 3H); ^13^C NMR (75 MHz, CD_3_OD) δ 172.1, 170.7, 137.8, 128.0, 127.5, 127.4, 72.8, 69.3, 52.9, 21.1; ESI-MS (*m/z*): calc. for C_12_H_15_NO_4_+Na [M+Na]^+^ 260.1, expr. 260.1.

### (*S*)-3-(benzyloxy)-2-pivalamidopropanoic acid (4e)

Using a similar procedure as described in **4c** with pivalic anhydride as a reagent, compound **4e** was obtained as a colorless oil (230.7 mg, 82.6%). ^1^H NMR (300 MHz, CD_3_OD) δ 7.31 (m, 5H), 4.64 (t, *J* = 6.0 Hz, 1H), 4.52 (s, 2H), 3.78 (dd, *J* = 6.0, 3.0 Hz, 2H), 1.20 (s, 9H); ^13^C NMR (75 MHz, CD_3_OD) δ 179.6, 170.8, 137.8, 128.1, 127.6, 127.5, 72.8, 69.1, 52.8, 38.3, 26.4; ESI-MS (*m/z*): calc. for C_15_H_21_NO_4_+Na [M+Na]^+^ 302.1, expr. 302.4.

### (*R*)-3-(benzyloxy)-2-pivalamidopropanoic acid (4f)

Using a similar procedure as described in **4c** with pivalic anhydride as a reagent, compound **4f** was obtained as a colorless oil (227.7 mg, 81.5%). ^1^H NMR (300 MHz, CD_3_OD) δ 7.30 (m, 5H), 4.67 (t, *J* = 6.0 Hz, 1H), 4.50 (s, 2H), 3.93, 3.74 (dd, *J* = 6.0, 3.0 Hz, 2H), 1.20 (s, 9H); ^13^C NMR (75 MHz, CD_3_OD) δ 179.6, 170.8, 137.8, 128.2, 127.6, 127.6, 72.8, 69.1, 52.8, 38.4, 26.5; ESI-MS (*m/z*): calc. for C_15_H_21_NO_4_+Na [M+Na]^+^ 302.1, expr. 302.3.

### (*S*,*E*) - 1 - acetyl -*N*-(2-(2-cyano-3-(4-hydroxyphenyl)acrylamido)ethyl)pyrroli- dine-2-carboxamide (5a)

*N*-acetyl-*O*-benzyl-L-serine (79.0 mg, 0.5 mmol), HOBt (101.5 mg, 0.75 mmol), and EDC·HCl (144.0 mg, 0.75 mmol) were dissolved in DCM/DMF (10:1, *V/V*) (15 ml). The mixture was stirred at room temperature for 10 min, following the addtion of DIPEA (0.52 ml, 3.0 mmol). The mixture was named **A** solution. Afterwards, **A** solution was added to a solution of compound **3a** (150.0 mg, 0.55 mmol) in DCM/DMF (10:1, *V/V*) (10 ml) dropwise via syringe at −30 °C with stirring. The temperature was then slowly warmed up to room temperature. The reaction mixture was stirred for 24 h. After removal of excess solvent, the residue was purified by column chromatography over silica gel using EtOAc/petroleum (1:1, *V/V*), to afford the slightly brown sticky oil **5a** (138.9 mg, yield: 68.2%). 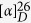 −83.2 (c = 1.0, MeOH). ^1^H NMR (300 MHz, CD_3_OD) δ 8.07 (s, 1H), 7.91 (d, *J* = 9.0 Hz, 2H), 6.93 (d, *J* = 9.0 Hz, 2H), 4.37 (m, 1H), 3.63 (m, 2H), 3.48 (t, *J* = 6.0 Hz, 2H), 3.40 (t, *J* = 6.0 Hz, 2H), 2.24 (m, 2H), 2.10 (s, 3H), 1.98 (m, 2H); ^13^C NMR (75 MHz, CD_3_OD) δ 173.9, 171.3, 163.3, 162.2, 151.3, 133.0, 123.4, 116.6, 115.8, 100.3, 60.3, 48.1, 39.7, 38.8, 29.9, 24.2, 21.1; ESI-MS (*m/z*): calc. for C_19_H_22_N_4_O_4_+Na [M+Na]^+^ 393.4, found 393.5; HRMS-ESI (*m/z*): calc. for C_19_H_22_N_4_O_4_+H [M+H]^+^ 371.1719, expr. 371.1714. HPLC purity: 96.2%.

### (*R*,*E*) -1-acetyl - *N* - (2-(2-cyano-3-(4-hydroxyphenyl)acrylamido)ethyl)pyrroli- dine-2-carboxamide (5b)

Using a similar procedure as described in **5a**, compound **5b** was obtained as a slightly brown sticky oil (137.1 mg, yield: 67.3%). 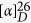 +81.5 (c = 1.0, MeOH). ^1^H NMR (300 MHz, CD_3_OD) δ 8.06 (s, 1H), 7.90 (d, *J* = 9.0 Hz, 2H), 6.93 (d, *J* = 9.0 Hz, 2H), 4.38 (m, 1H), 3.58 (m, 2H), 3.47 (t, *J* = 6.0 Hz, 2H), 3.41 (t, *J* = 6.0 Hz, 2H), 2.24 (m, 2H), 2.10 (s, 3H), 1.98 (m, 2H); ^13^C NMR (75 MHz, CD_3_OD) δ 173.9, 171.4, 163.4, 162.2, 151.4, 133.1, 123.4, 116.6, 115.9, 100.2, 60.3, 48.1, 39.7, 38.8, 29.7, 24.3, 21.1; ESI-MS (*m/z*): calc. for C_19_H_22_N_4_O_4_+Na [M+Na]^+^ 393.4, found 393.3; HRMS-ESI (*m/z*): calc. for C_19_H_22_N_4_O_4_+H [M+H]^+^ 371.1719, expr. 371.1714. HPLC purity: 97.5%.

### (*S*,*E*) – *N* - (2 - (2 -acetamido-3-(benzyloxy) propanamido) ethyl)-2-cyano-3-(4- hydroxyphenyl)acrylamide (5c)

Using a similar procedure as described in **5a**, compound **5c** was obtained as a slightly yellow solid (179.6 mg, yield: 72.5%). mp: 176–178 °C, 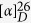 −9.3 (c = 1.0, MeOH). ^1^H NMR (300 MHz, DMSO-*d*_*6*_) δ 10.64 (s, 1H), 8.27 (s, 1H), 8.15 (d, *J* = 6.0 Hz, 1H), 8.13 (t, *J* = 6.0 Hz, 1H), 8.05 (t, *J* = 6.0 Hz, 1H), 7.90 (d, *J* = 9.0 Hz, 2H), 7.29 (m, 5H), 7.0 (d, *J* = 9.0 Hz, 2H), 4.52 (t, *J* = 6.0 Hz, 1H), 4.47 (s, 2H), 3.61 (dd, *J* = 3.0 Hz, 2H), 3.5 (s, 1H), 3.3 (m, 3H), 1.88 (s, 3H); ^13^C NMR (75 MHz, DMSO-*d*_*6*_) δ 170.5, 170.0, 162.3, 162.2, 150.9, 138.6, 133.3, 128.6, 127.9, 123.4, 117.6, 116.7, 101.6, 72.4, 70.3, 53.3, 38.7, 23.0; ESI-MS (*m/z*): calc. for C_24_H_26_N_4_O_5_+Na [M+Na]^+^ 473.2, found 473.5; HRMS-ESI (*m/z*): calc. for C_24_H_26_N_4_O_5_+H [M+H]^+^ 451.1981, expr. 451.1977. HPLC purity: 98.3%.

### (*R*,*E*) – *N* - (2 - (2 -acetamido-3-(benzyloxy) propanamido) ethyl)-2-cyano-3-(4- hydroxyphenyl)acrylamide (5d)

Using a similar procedure as described in **5a**, compound **5d** was obtained as a slightly yellow solid (179.1 mg, yield: 72.3%). mp: 175–177 °C, 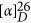 +8.7 (c = 1.0, MeOH). ^1^H NMR (300 MHz, DMSO-*d*_*6*_) δ 10.62 (s, 1H), 8.26 (s, 1H), 8.14 (d, *J* = 6.0 Hz, 1H), 8.11 (t, *J* = 6.0 Hz, 1H), 8.04 (t, *J* = 6.0 Hz, 1H), 7.89 (d, *J* = 9.0 Hz, 2H), 7.29 (m, 5H), 6.96 (d, *J* = 9.0 Hz, 2H) 4.50 (t, *J* = 6.0 Hz, 1H), 4.47 (s, 2H), 3.60 (dd, *J* = 3.0 Hz, 2H), 3.27 (t, *J* = 6.0 Hz, 4H), 1.88 (s, 3H); ^13^C NMR (75 MHz, DMSO-d_6_) δ 170.4, 170.0, 162.3, 162.2, 150.9, 138.6, 133.3, 128.6, 127.9, 123.4, 117.6, 116.7, 101.5, 72.4, 70.3, 50.3, 38.7, 23.0; ESI-MS (*m/z*): calc. for C_24_H_26_N_4_O_5_+Na [M+Na]^+^ 473.2, found 473.4; HRMS-ESI (*m/z*): calc. for C_24_H_26_N_4_O_5_+H [M+H]^+^ 451.1981, expr. 451.1976. HPLC purity: 97.8%.

### (*S*,*E*) – *N* - (3 -(2-acetamido-3-(benzyloxy) propanamido) propyl)-2-cyano-3-(4- hydroxyphenyl)acrylamide (5e)

Using a similar procedure as described in **5a**, compound **5e** was obtained as a yellow sticky oil (190.6 mg, yield: 74.6%). 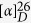 −8.6 (c = 1.0, MeOH). ^1^H NMR (300 MHz, DMSO-*d*_*6*_) δ 10.62 (s, 1H), 8.25 (s, 1H), 8.15 (d, *J* = 6.0 Hz, 1H), 8.06 (t, *J* = 6.0 Hz, 1H), 8.04 (t, *J* = 6.0 Hz, 1H), 7.90 (d, *J* = 9.0 Hz, 2H), 7.30 (m, 5H), 6.96 (d, *J* = 9.0 Hz, 2H), 4.48 (m, 3H), 3.60 (dd, *J* = 3.0 Hz, 2H), 3.18 (m, 2H), 3.13 (m, 2H), 1.89 (s, 3H), 1.68 (m, 2H); ^13^C NMR (75 MHz, DMSO-*d*_*6*_) δ 170.2, 170.0, 162.2, 162.0, 150.9, 138.6, 133.3, 128.6, 127.9, 127.9, 123.4, 117.7, 116.9, 101.6, 72.5, 70.3, 56.5, 37.7, 36.9, 29.4, 23.0; ESI-MS (*m/z*): calc. for C_25_H_28_N_4_O_5_+Na [M+Na]^+^ 487.2, expr. 487.5; HRMS-ESI (*m/z*): calc. for C_25_H_28_N_4_O_5_+H [M+H]^+^ 465.2138, found 465.2133. HPLC purity: 96.5%.

### (*R*,*E*) - *N* - (3-(2-acetamido-3-(benzyloxy) propanamido) propyl)-2-cyano-3-(4- hydroxyphenyl)acrylamide (5f)

Using a similar procedure as described in **5a**, compound **5f** was obtained as a yellow solid (191.9 mg, yield: 75.1%). mp: 181–183 °C, 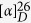 +7.8 (c = 1.0, MeOH). ^1^H NMR (300 MHz, DMSO-d_6_) δ 10.60 (s, 1H), 8.28 (s, 1H), 8.15 (d, *J* = 6.0 Hz, 1H), 8.09 (t, *J* = 6.0 Hz, 1H), 8.04 (t, *J* = 6.0 Hz, 1H), 7.92 (d, *J* = 9.0 Hz, 2H), 7.30 (m, 5H), 6.98 (d, *J* = 9.0 Hz, 2H), 4.49 (m, 3H), 3.63 (dd, *J* = 3.0 Hz, 2H), 3.46 (m, 1H), 3.18 (m, 2H), 3.13 (m, 2H), 1.89 (s, 3H), 1.68 (m, 2H); ^13^C NMR (75MHz, DMSO-d_6_) δ 170.2, 169.9, 162.3, 161.9, 150.9, 138.6, 133.3, 128.6, 127.9, 123.4, 117.7, 116.7, 101.5, 72.5, 70.3, 53.3, 37.7, 36.9, 29.4, 23.0; ESI-MS (*m/z*): calc. for C_25_H_28_N_4_O_5_+Na [M+Na]^+^ 487.2, found 487.5; HRMS-ESI (*m/z*): calc. for C_25_H_28_N_4_O_5_+H [M+H]^+^ 465.2138, expr. 465.2132. HPLC purity: 97.8%.

### (*S*,*E*) - *N* - (2-(2-pivalamido-3-(benzyloxy) propanamido) ethyl)-2-cyano-3-(4- hydroxyphenyl)acrylamide (5g)

Using a similar procedure as described in **5a**, compound **5g** was obtained as a yellow oil (182.1 mg, yield: 67.2%). 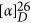 −10.2 (c = 1.0, MeOH). ^1^H NMR (300 MHz, CD_3_OD) δ 8.06 (s, 1H), 7.89 (d, *J* = 9.0 Hz, 2H), 7.29 (m, 5H), 6.93 (d, *J* = 9.0 Hz, 2H), 4.60 (t, *J* = 6.0 Hz 1H), 4.52 (s, 2H), 3.75 (m, 2H), 3.47 (m, 4H), 1.21 (s, 9H); ^13^C NMR (75MHz, CD_3_OD) δ 179.9, 171.5, 163.2, 162.2, 151.6, 137.8, 133.1, 128.1, 127.6, 127.5, 123.4, 116.6, 115.9, 100.0, 72.7, 69.3, 53.4, 39.8, 38.9, 38.4, 26.4; ESI-MS (*m/z*): calc. for C_27_H_32_N_4_O_5_+Na [M+Na]^+^ 515.2, found 515.5; HRMS-ESI (*m/z*): calc. for C_27_H_32_N_4_O_5_+H [M+H]^+^ 493.2451, expr. 493.2445. HPLC purity: 98.1%.

### (*R*,*E*) - *N* - (2-(2-pivalamido-3-(benzyloxy) propanamido) ethyl)-2-cyano-3-(4- hydroxyphenyl)acrylamide (5h)

Using a similar procedure as described in **5a**, compound **5h** was obtained as a yellow oil (185.1 mg, yield: 68.3%). 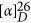 +9.4 (c = 1.0, MeOH). ^1^H NMR (300 MHz, CD_3_OD) δ 8.15 (s, 1H), 7.95 (d, *J* = 9.0 Hz, 2H), 7.37 (m, 5H), 7.01 (d, *J* = 9.0 Hz, 2H), 4.67 (t, *J* = 6.0 Hz 1H), 4.60 (s, 2H), 3.81 (m, 2H), 3.54 (m, 4H), 1.29 (s, 9H); ^13^C NMR (75 MHz, CD_3_OD) δ 179.0, 170.6, 162.4, 161.4, 150.7, 137.0, 132.3, 127.3, 126.8, 126.7, 122.6, 115.8, 115.1, 99.2, 71.9, 68.5, 52.6, 39.0, 38.1, 37.6, 25.6; ESI-MS (*m/z*): calc. for C_27_H_32_N_4_O_5_+Na [M+Na]^+^ 515.2, found 515.5; HRMS-ESI (*m/z*): calc. for C_27_H_32_N_4_O_5_+H [M+H]^+^ 493.2451, expr. 493.2445. HPLC purity: 98.3%.

### (*S*,*E*) - *N* - (3-(2-pivalamido-3-(benzyloxy) propanamido) propyl)-2-cyano-3-(4- hydroxyphenyl)acrylamide (5i)

Using a similar procedure as described in **5a**, compound **5i** was obtained as a yellow sticky oil (185.3 mg, yield: 66.5%). 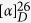 −11.3 (c = 1.0, MeOH). ^1^H NMR (300 MHz, CD_3_OD) δ 8.07 (s, 1H), 7.90 (d, *J* = 9.0 Hz, 2H), 7.33 (m, 5H), 6.93 (d, *J* = 9.0 Hz, 2H), 4.57 (t, *J* = 6.0 Hz 1H), 4.54 (s, 2H), 3.78 (dd, *J* = 6.0 Hz, 2H), 3.32 (t, *J* = 6.0 Hz 2H), 3.27 (m, 2H), 1.78 (m, 2H), 1.22 (s, 9H); ^13^C NMR (75 MHz, CD_3_OD) δ 179.8, 170.1, 162.8, 162.2, 151.4, 137.8, 133.0, 128.1, 127.6, 127.5, 123.4, 116.6, 115.8, 100.1, 72.8, 69.3, 53.5, 38.4, 37.0, 36.2, 28.8, 26.3; ESI-MS (*m/z*): calc. for C_28_H_34_N_4_O_5_+Na [M+Na]^+^ 529.4, expr. 529.5; HRMS-ESI (*m/z*): calc. for C_28_H_34_N_4_O_5_+H [M+H]^+^ 507.2607, expr. 507.2602. HPLC purity: 97.6%.

### (*R*,*E*) - *N* - (3-(2-pivalamido-3-(benzyloxy) propanamido) propyl)-2-cyano-3-(4- hydroxyphenyl)acrylamide (5j)

Using a similar procedure as described in **5a**, compound **5j** was obtained as a yellow sticky oil (185.9 mg, yield: 66.7%). 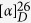 +10.7 (c = 1.0, MeOH). ^1^H NMR (300 MHz, CD_3_OD) δ 8.10 (t, *J* = 6.0 Hz 1H), 8.07 (s, 1H), 7.89 (d, *J* = 9Hz, 2H), 7.31 (m, 5H), 6.93 (d, *J* = 9.0 Hz, 2H), 4.57 (t, *J* = 6.0 Hz, 1H), 4.53 (s, 2H), 3.76 (dd, *J* = 6.0 Hz, 2H), 3.34 (t, 2H), 3.27 (m, 2H), 1.76 (m, 2H), 1.22 (s, 9H); ^13^C NMR (75 MHz, CD_3_OD) δ 179.9, 171.1, 162.8, 162.2, 151.4, 137.8, 133.1, 128.1, 127.6, 127.5, 123.4, 116.6, 115.9, 100.1, 72.8, 69.3, 53.6, 38.5, 37.0, 36.2, 28.9, 26.4; ESI-MS (*m/z*): calc. for C_28_H_34_N_4_O_5_+Na [M+Na]^+^ 529.4, expr. 529.4; HRMS-ESI (*m/z*): calc. for C_28_H_34_N_4_O_5_+H [M+H]^+^ 507.2607, expr. 507.2606. HPLC purity: 97.3%.

### Aldose reductase inhibition

Human ALR2 was purchased from WAKO Pure Chemical Industries, Ltd. (Japan), which was purified from the culture medium of baculovirus-insect cell expression system. Epalrestat was used as a positive reference. ALR2 inhibition was determined spectrophotometrically according to the procedure described previously^52^.

### ORAC_FL_ assay

Antioxidant capacity was determined according to the procedure described previously^41^,^53^.

### Molecular docking

Molecular docking was carried out using Autodock/Vina^54^ with the PyRx virtual screening graphical interface. The crystal structure of human aldose reductase in complex with NADP and IDD type inhibitor (pdbcode 2IKI) was used as a macromolecule for docking studies. Prior to the docking, water molecules and the bound ligands were deleted from the macromolecule. Both the macromolecule and small molecule ligands were processed by Autodock Tools (ADT) as pdbqt format, a special PDB format with charge and atom type, and additional topological information of rotatable bonds for ligands. In order to take all the possible binding sites into consideration, a maximized grid map was generated for all the atom types.

### Stability

The sample was incubated in high-glucose Dulbecco’s modified Eagle’s medium (DMEM) containing 5% fetal calf serum (FBS) for a period of 48 h. Analyses were scheduled at 0.5, 1.0, 4.0, 8.0, 12.0, 24.0, and 48.0 h. Reverse-phase high performance liquid chromatography (RP-HPLC) was performed on a Cosmosil column (C_18_, 5 Å, 4.6 × 250 mm) using an LC-100 fluid unit (Shanghai Wufeng Inc., China) with a tunable LC-100 UV detector. The eluent was various percentages of methanol containing pure water (flow rate: 1.0 ml/min; λ: 254 nm). The experiments were repeated at least 3 times and compared with the control experiment. The results were shown in Supporting Infromation.

### Cytotoxicity

HEK293 cells were purchased from Centre of Cells Resource, Shanghai Institute of Life Science, Chinese Academy of Sciences, P. R. China. HEK293 cells in the exponential phase were plated on a 96-well cell culture plate with 5 × 10^3^ cells in each well supplemented with 100 U/ml of penicillin and 100 μg/ml of streptomycin, which were cultured in a humidified atmosphere of 95% and 5% CO_2_ at 37 °C for 24 h. The cells were adherent. Testing samples and epalrestat at final concentrations of 5.0, 50, 500 nM, and 5.0 and 500 μM, respectively, were added. 6 wells were set for each group. The wells without addition of the drug were set as controls. The cells in each of the wells were cultured for another 24 h. Then 15 μl of MTT (5 mg/mL) was added to each well. The plates were then incubated at 37 °C for 4 hours, then the supernatants were discarded. 150 μL of DMSO solutions were added to each of the wells and the solutions were mixed thoroughly. Then the plates were incubated at 37 °C for another 10 minutes. Each sample was mixed again and the resulting formazan was measured by its absorbance at 570 nm using a BIO-RAD680 plate reader (Thermo, USA). The experiments were repeated at least 3 times and compared with the control experiment. The results were shown in Supporting Infromation.

### Mortality, NTD rate and body weight measurements

Fertilized leghorn eggs were purchased from the Avian Farm of the South China Agriculture University (Guangzhou, China). Before the injection of glucose (Sigma–Aldrich, MO, USA), the eggs were incubated in a humidified incubator (Grumbach, Germany) at 37 °C with 60% humidity until the embryos reached the desired stage. Embryos were divided into 7 groups (n = 20, each group), including control, glucose (0.4 mmol/egg), three dosages of **5f** groups (CL, 10 nmol/egg; CM, 100 nmol/egg; and CH, 1 μmol/egg), epalrestat (1 μmol/egg), and edaravone (0.1 nmol/egg). **5f**, epalrestat and edaravone were dissolved in 100 μL chicken saline (0.72% NaCl) and injected into the air sac on EDD 0, respectively. Glucose was dissolved in 200 μL chicken saline and injected on EDD 1. Mortality, abnormality and body weight measurements were made on EDD 5. Embryos were removed from their eggs and weighed. The percent mortality was measured by counting the number of dead versus all embryos. The rate of NTD of the embryos was assessed by the number of NTD versus survival embryos.

### Plasma glucose measurement

The ventricles of embryos were punctured with a 20-gauge needle. At this early embryonic age, 30–40 μL of blood was obtained from the embryos on each EDD 5. The blood containing heparin was centrifuged at 3000 rpm for 15 min. The plasma glucose concentration was measured using a glucose oxidase-coupled spectrophotometric assay kit obtained from Sigma Chemical Co. (St. Louis, MO, USA) according to the manufacturer’s instructions.

### Histological analysis

The whole-mount embryos on EDD 5 were photographed using a stereo-fluorescence microscope (Olympus MVX10) and processed by the Olympus software package Image-Pro Plus 7.0. Further histological examination was performed by using hematoxylin and eosin (H&E) staining. In brief, embryos were immersed in 4% paraformaldehyde for 3 d, processed for paraffin embedding, and sectioned at 5 μm thickness. Sections were then processed for H&E staining as per standard histological protocols. The results were shown in Supporting Infromation.

### Measurement of AR activity and sorbitol content

Embryos on EDD 5 were homogenized with chicken saline and centrifuged (4 °C, 10000 rpm, 10 min). The supernatant was used for measuring AR activity in the sample. A chicken AR ELISA kit (Jianglai Biotechnology, China) were used for detection. The concentration of AR in the samples was then determined by comparing the OD of the samples to the standard curve. The sorbitol contents of the chick embryo were determined by a Sorbitol Colorimetric Assay Kit (BioVision, USA). In the assay, sorbitol is oxidized to fructose with the proportional development of intense color with an absorbance maximum at 560 nm.

### Measurement of ALR1 activity

Embryos on EDD 5 were homogenized with chicken saline and centrifuged (4 °C, 10000 rpm, 10 min). The supernatant was used for measuring ALR1 activity in the sample. A chicken ALR1 ELISA kit (Jianglai Biotechnology, China) were used for detection. The concentration of ALR1 in the samples was then determined by comparing the OD of the samples to the standard curve.

### Measurement of ROS generation ratio, MDA contents, ORAC level and in chick embryo eyes

ROS generation ratios in the eyes of EDD 5 chick embryos were detected with 5 μmol 2′,7′-dichlorofluorescein-diacetate (DCFH-DA, Sigma-Aldrich). The peroxide content was determined by measuring thiobarbituric-acid-reactive substances (TBARS) with a commercial MDA kit (Nanjing Jiancheng Institute of Biotechnology, Nanjing, China). The procedures for the ORAC assay were modified from the previously described method^55^. The automated ORAC assay was carried out on a decay of the fluorescein signal and determined with an excitation/emission filter pair of 485/527 nm in a GENios Lueifcrase microplate reader (TECAN) at 37 °C. Final results were calculated on the basis of the difference in the area under the fluorescein decay curve between the blank and each sample.

### Western blotting analysis

The eyes of EDD 5 chick embryos were separated and lysed in lysis buffer (Beyotime Institute of Biotechnology, China) on ice for 10 min. After centrifugation of the cell suspension at 12000 rpm for 15 min, the protein content of the supernatant was determined with a Pierce BCA protein assay kit (Thermo Fisher Scientific, USA) to ensure equal sample loading. Protein lysates were separated in 12% SDS-PAGE and blotted onto nitrocellulose membranes (Amersham Biosciences, USA). Proteins were detected using the monoclonal antibody anti-Pax3 diluted 1:1000 (DSHB, USA) and visualized using anti-mouse IgG conjugated with horseradish peroxidase (HRP) and Pierce ECL Western Blotting Substrate (Thermo Fisher Scientific, USA) as the substrate of HRP.

### Statistical Analysis

Experimental values were given as means ± SD. Statistical analysis of the data was performed using the SPSS 18.0 statistical software. One-way analysis of variance (ANOVA) was applied to analyze for difference in data of biochemical parameters among the different groups, followed by Dunnett’s significant post-hoc test for pairwise multiple comparisons. Differences were considered as statistically significant at *P < 0.05, **P < 0.01.

## Additional Information

**How to cite this article**: Zhang, L. *et al*. Bioactivity Focus of α-Cyano-4-hydroxycinnamic acid (CHCA) Leads to Effective Multifunctional Aldose Reductase Inhibitors. *Sci. Rep.* **6**, 24942; doi: 10.1038/srep24942 (2016).

## Supplementary Material

Supplementary Information

## Figures and Tables

**Figure 1:**
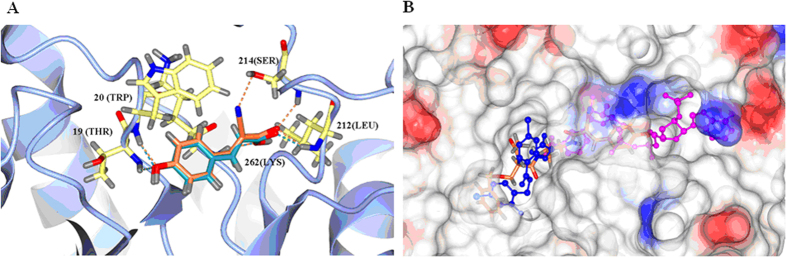
New design based on the docking study. (**A**) Superimposition of *p*-coumaric acid and CHCA in the active pocket. Dark cyan: *p*-coumaric acid; Coral: CHCA. (**B**) New suggested entity that occupy both the NADP^+^ and inhibitor binding sites. Blue: IDD inhibitor; Magenta: NADP^+^; Coral: new hypothetic entity. Molecular docking was carried out using Autodock/Vina (Trott and Olson^54^) with the PyRx virtual screening graphical interface. The crystal structure of human aldose reductase in complex with NADP and IDD type inhibitor (PDB code: 2IKI) was used as a macromolecule for docking studies.

**Table 1:** ALR2 inhibition activities, binding properties, and antioxidant capacities of compounds 5a–j.

Compd.	IC_50_ (nM)	Biding affinity (kcal/mol)	Docking score	ORAC (U/ml)		
				12.5 (μg/ml)	6.25 (μg/ml)	3.125 (μg/ml)
**5a**	137.1 ± 2.9	−10.6	−11.675	49.8 ± 1.7	52.4 ± 2.1	57.0 ± 2.3
**5b**	135.9 ± 2.4	−11.8	−11.744	42.9 ± 3.3	44.1 ± 2.5	47.1 ± 2.4
**5c**	315.7 ± 6.2	−9.4	−9.037	26.4 ± 2.9	30.4 ± 1.7	32.4 ± 1.8
**5d**	404.4 ± 7.0	−8.3	−7.208	33.3 ± 4.1	35.4 ± 3.2	38.3 ± 1.9
**5e**	99.8 ± 2.0	−12.1	−11.845	60.4 ± 2.2	62.6 ± 1.5	65.7 ± 2.7
**5f**	72.7 ± 1.6	−12.3	−12.859	65.7 ± 5.1	66.5 ± 1.6	68.1 ± 2.3
**5g**	272.5 ± 4.6	−9.9	−9.775	30.4 ± 3.7	33.9 ± 1.4	38.3 ± 2.6
**5h**	251.6 ± 4.6	−9.4	−10.097	42.8 ± 1.1	45.2 ± 1.3	50.3 ± 3.2
**5i**	220.7 ± 3.9	−10.0	−11.528	35.3 ± 2.1	35.6 ± 3.9	36.4 ± 3.5
**5j**	246.6 ± 4.5	−11.1	−10.167	30.6 ± 2.2	35.3 ± 1.7	38.6 ± 1.9
**CHCA**	132.7 ± 1.8	–	–	48.8 ± 1.3	51.2 ± 1.1	52.3 ± 3.1
**Mangiferin**	138.0 ± 2.2	–	–	73.2 ± 7.2	74.8 ± 0.8	75.3 ± 0.4
**Trolox**	–	–	–	85.2 ± 4.3	92.8 ± 2.7	95.3 ± 3.7
**Epalrestat**	61.3 ± 1.3	–	–	–	–	–

**Table 2:** Effects of normal, sham, control (saline), **5f**, epalrestat and edaravone treatments on the percentage of embryo death, NTD and body weight.

**Items**	**Treatment (n = 20)**						
	**Control**	**Glucose (0.4 mmol/egg)**					
		**Model**	**CL 10 nM**	**CM 100 nM**	**CH 1 μM**	**Epalrestat 1 μM**	**Edaravone 0.1 nM**
Death (/n)	8.7%	40.0%	35.0%	30.0%	25.0%	25.0%	20.0%
NTD (/survival)	1.6%	58.3%	46.20%	35.7%	20.0%	26.7%	18.8%
Body weight (mg)	310.4 ± 6.3	286.2 ± 8.5**	291.3 ± 10.2	295.9 ± 13.2#	307.9 ± 10.5##	300.1 ± 3.6#	310.8 ± 11.6##

*P < 0.05, **P < 0.01 *vs*. control.
