# Supplementary material for: Bioactivity Focus of α-Cyano-4-hydroxycinnamic acid (CHCA) Leads to Effective Multifunctional Aldose Reductase Inhibitors
Source: Sci Rep. 2016 Apr 25;6:24942. doi: 10.1038/srep24942 (PMC4842970; doi:10.1038/srep24942)
Supplement: Supplementary Information [file srep24942-s1.pdf]

Supporting information

Bioactivity Focus on α-Cyano-4-hydroxycinnamic acid (CHCA) Leads to Effective Multifunctional Aldose Reductase Inhibitors

Laitao Zhang, [†,a] Yifang Li, [†,§,a] Sheng Yuan,[†,a] Shijie Zhang,[⊥] Huanhuan Zheng,[†] Jie Liu,[†] Pinghua Sun,[†] Yijun Gu,[‖] Hiroshi Kurihara,[†,§] Rongrong He,*[,†,§] and Heru Chen*[,†,‡,§]

[†]Institute of Traditional Chinese Medicine and Natural Product College of Pharmacy, Jinan University Guangzhou 510632, P. R. China

[‡]State key Laboratory of Drug Research, Shanghai Institute of Materia Medica, Chinese Academy of Sciences, Shanghai 201203, P. R. China

[⊥]Institute of Clinical Pharmacology, Guangzhou University of Chinese Medicine, Guangzhou 510006, P.R. China

[‖]Institute of Biochemistry and Cell Biology, Chinese Academy of Sciences, Shanghai 200031, P. R. China

[§]Guangdong Province Key Laboratory of Pharmacodynamic Constituents of TCM and New Drugs Research, Guangzhou 510632, P. R. China

correspondence should be directed to thrchen@jnu.edu.cn

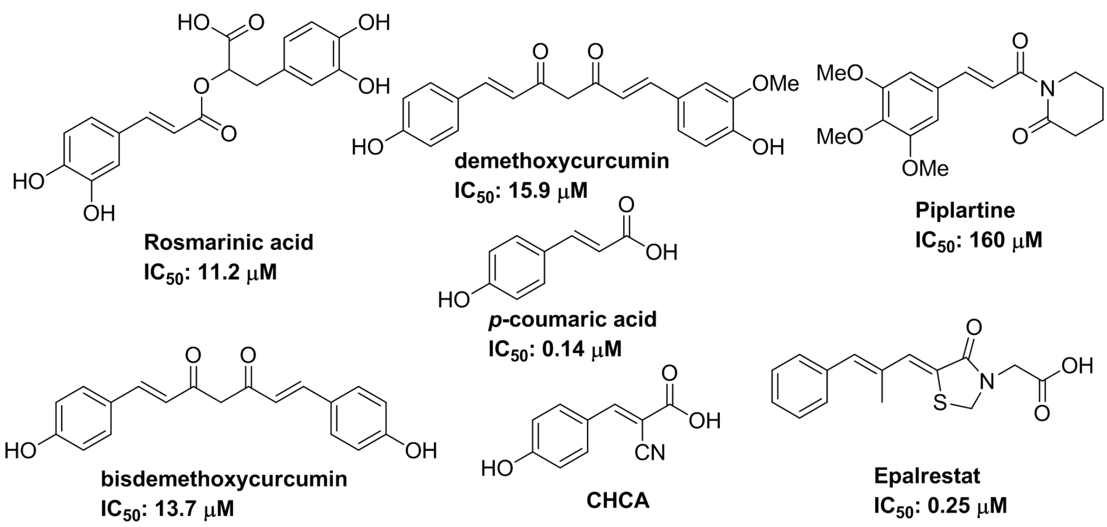

Fig. S1. Chemical structures of some representative ARIs.

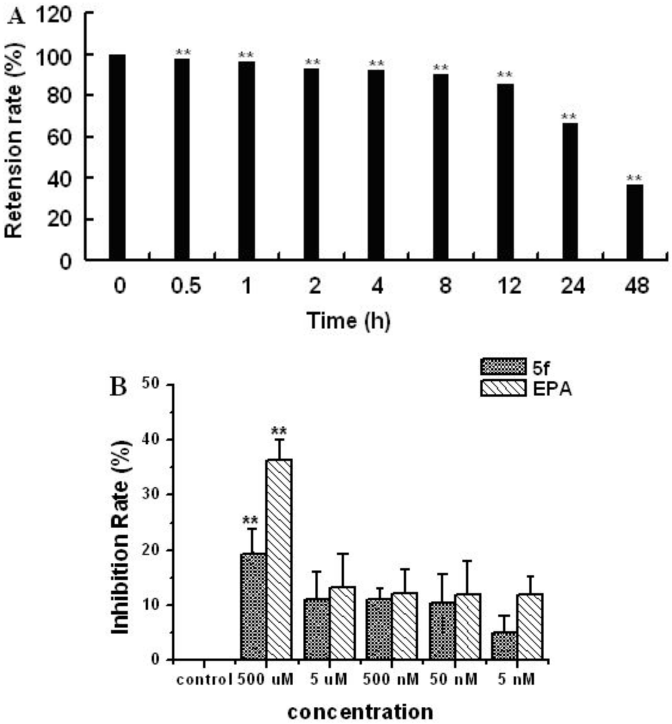

Fig. S2. Stability and toxicity of **5f**. (**A**) Stability studies with **5f** incubated in 5% FBS DMEM showing % retention over time. RP-HPLC was used as the assay. Analyses were scheduled at 0.5, 1.0, 4.0, 8.0, 12.0, 24.0, and 48.0 h. $^{**}P \leq 0.01$ *vs* control group. (**B**) Growth inhibition rate of **5f** and EPS against HEK293 cells. MTT assay was used to determine the cell viability. Testing samples and epalrestat at final concentrations of 5.0, 50, 500 nM, and 5.0, 50, and 500 μM, respectively, were assessed $^{**}P \leq 0.01$ *vs* control group.

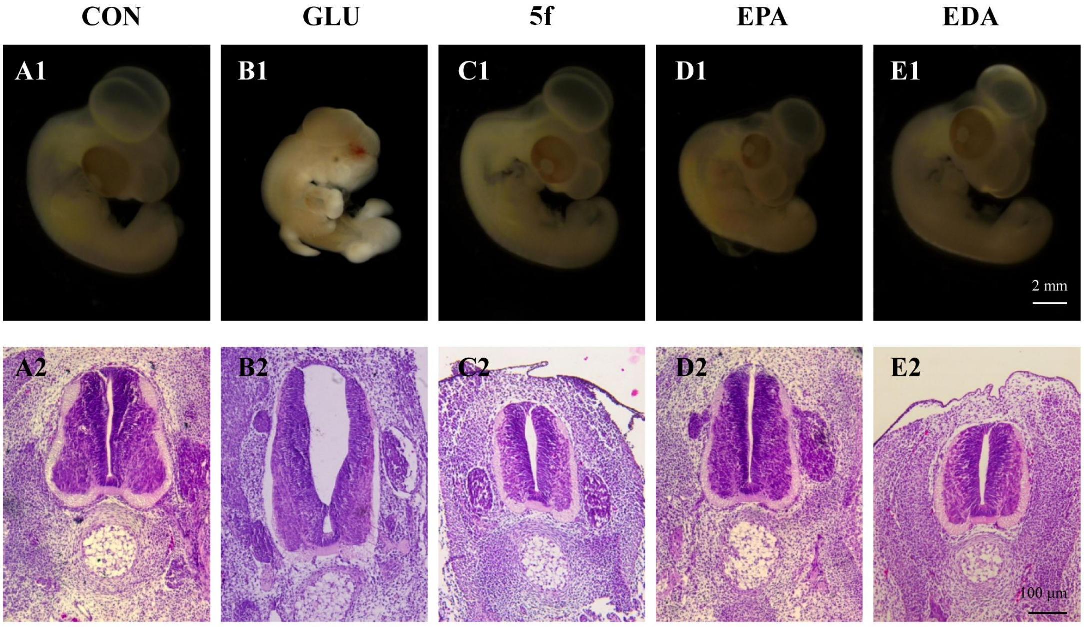

Fig. S3. 5f ameliorated high-glucose-induced NTD in chick embryos on EDD 5. Stereoscopic microscope measurement of whole body and Hematoxylin and eosin (H&E) staining of embryo sections. Scale bars of **A1-E1**: 2 mm. Scale bars of **A2-E2**: 100 μm.

II. The NMR and HRMS data of compounds 5a-j are listed in the following

5a (^{1}H NMR, ^{13}C NMR, HRMS)

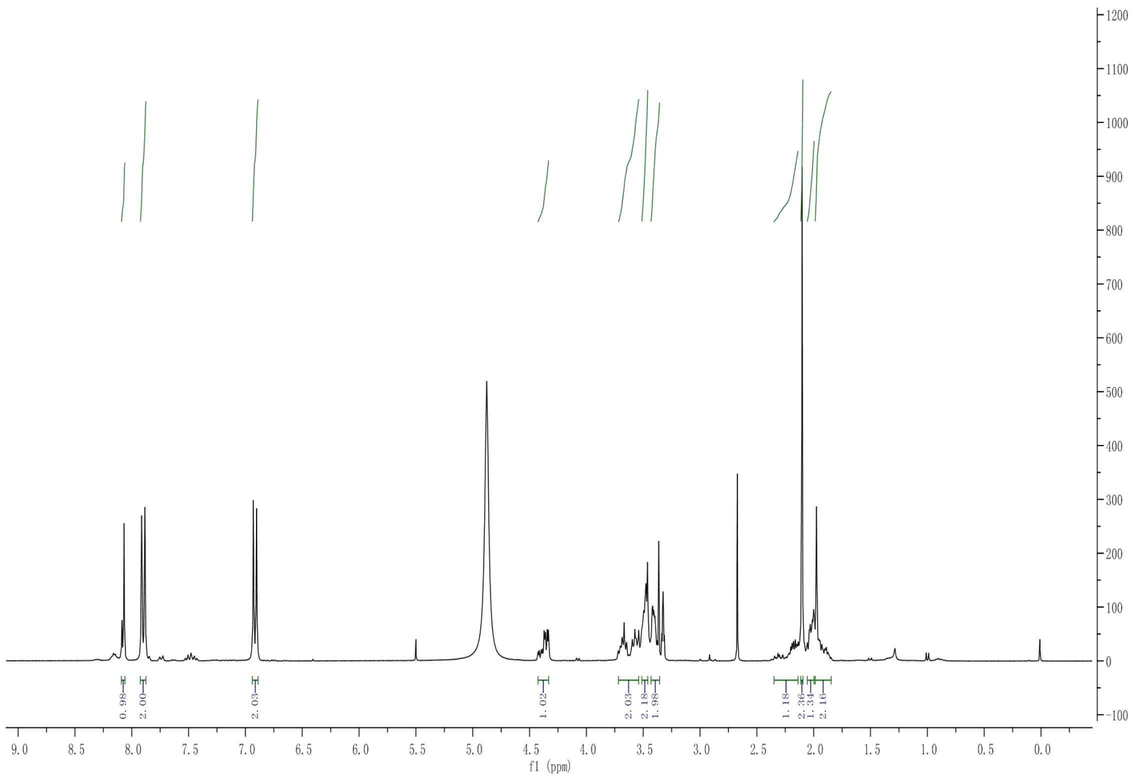

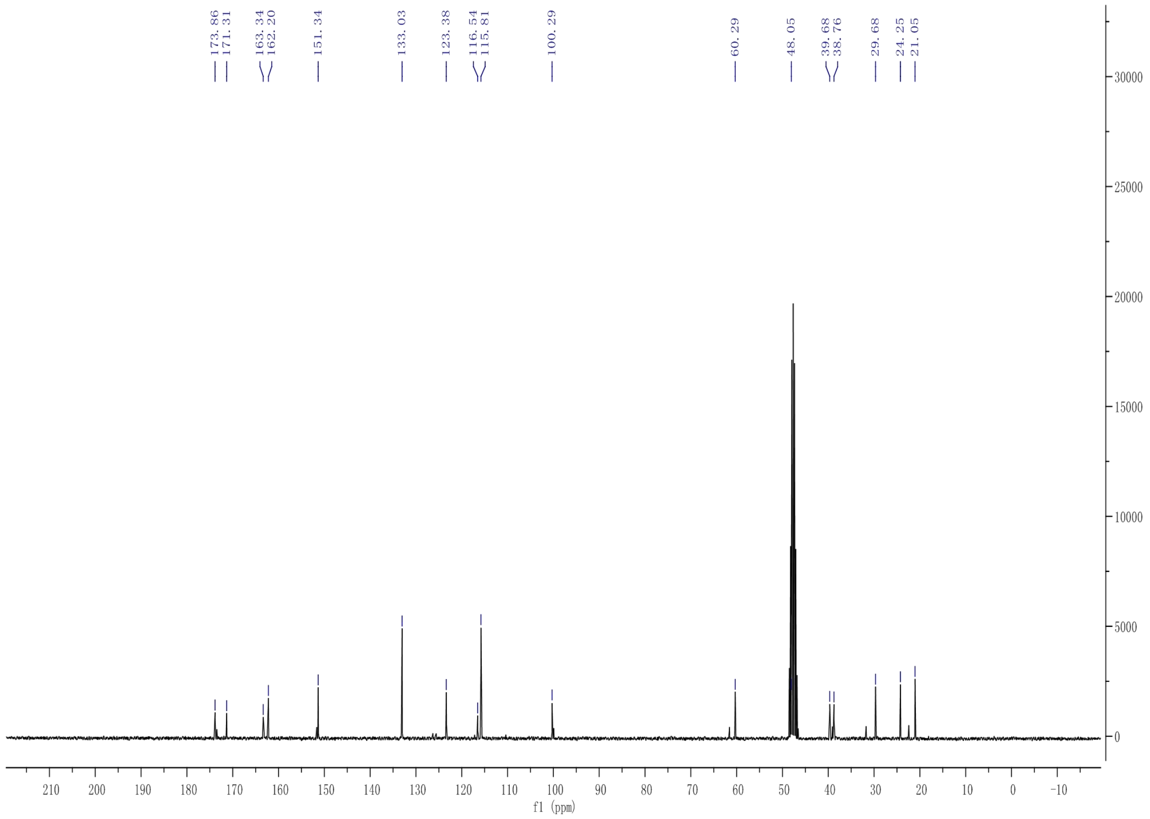

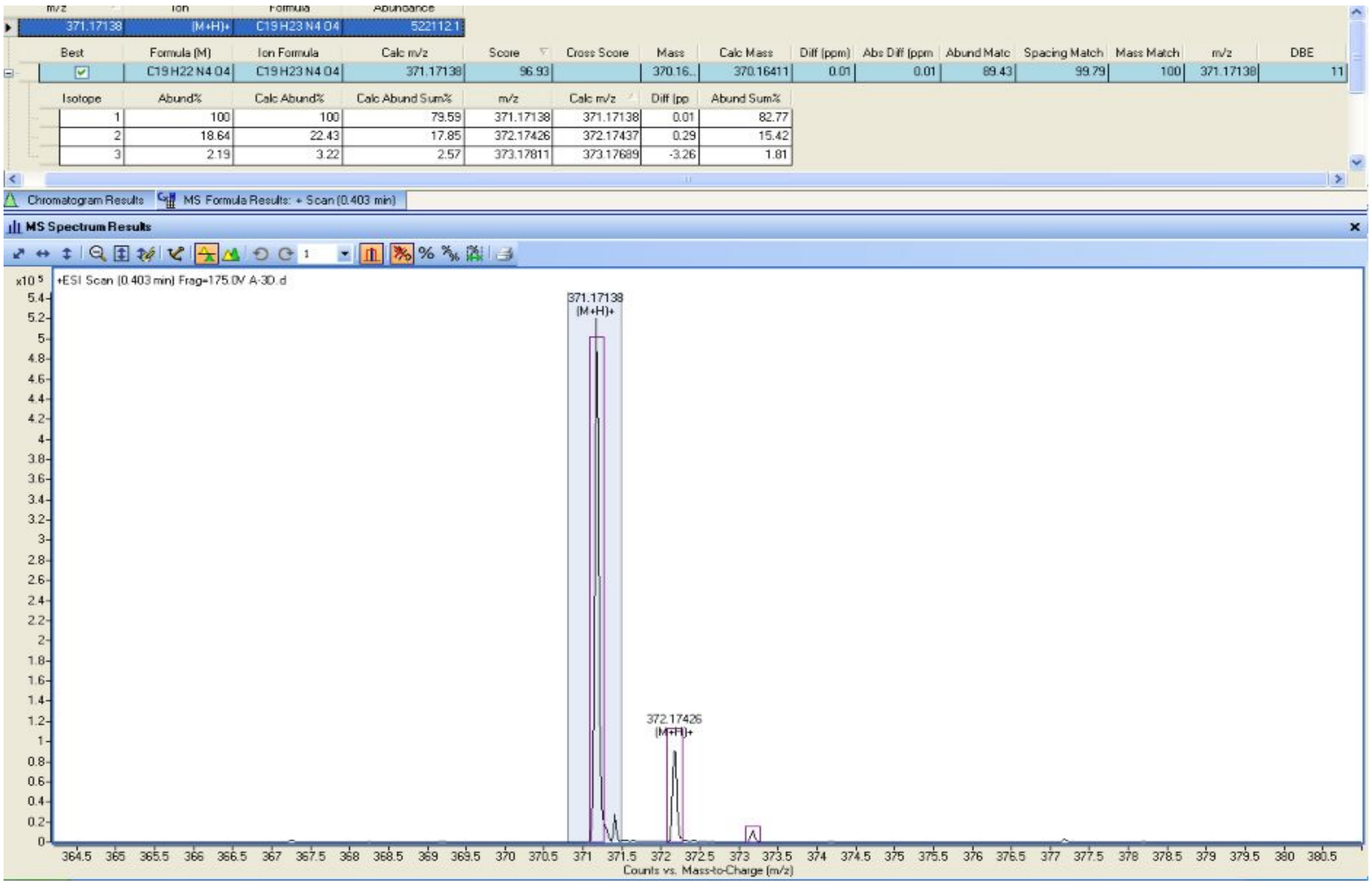
m/z Ion Formula Abundance
371.17138 (M+H)+ C19H23 N4 O4 522112.1
Best Formula (M) Ion Formula Calc m/z Score Cross Score Mass Calc Mass Diff (ppm) Abs Diff (ppm Abund Matc Spacing Match Mass Match m/z DBE
C19H22 N4 O4 C19H23 N4 O4 371.17138 96.93 370.16.. 370.16411 0.01 0.01 89.43 99.79 100 371.17138 11
Isotope Abund% Calc Abund% Calc Abund Sum% m/z Calc m/z Diff (pp Abund Sum%
1 100 100 79.59 371.17138 371.17138 0.01 82.77
2 18.64 22.43 17.85 372.17426 372.17437 0.29 15.42
3 2.19 3.22 2.57 373.17811 373.17689 -3.26 1.81
Chromatogram Results
MS Formula Results: + Scan (0.403 min)
MS Spectrum Results
x10 5
+ESI Scan (0.403 min) Frag=175.0V A-30.d
371.17138
(M+H)+
372.17426
(M+H)+
Counts vs. Mass-to-Charge (m/z)

5b (^{1}H NMR, ^{13}C NMR, HRMS)

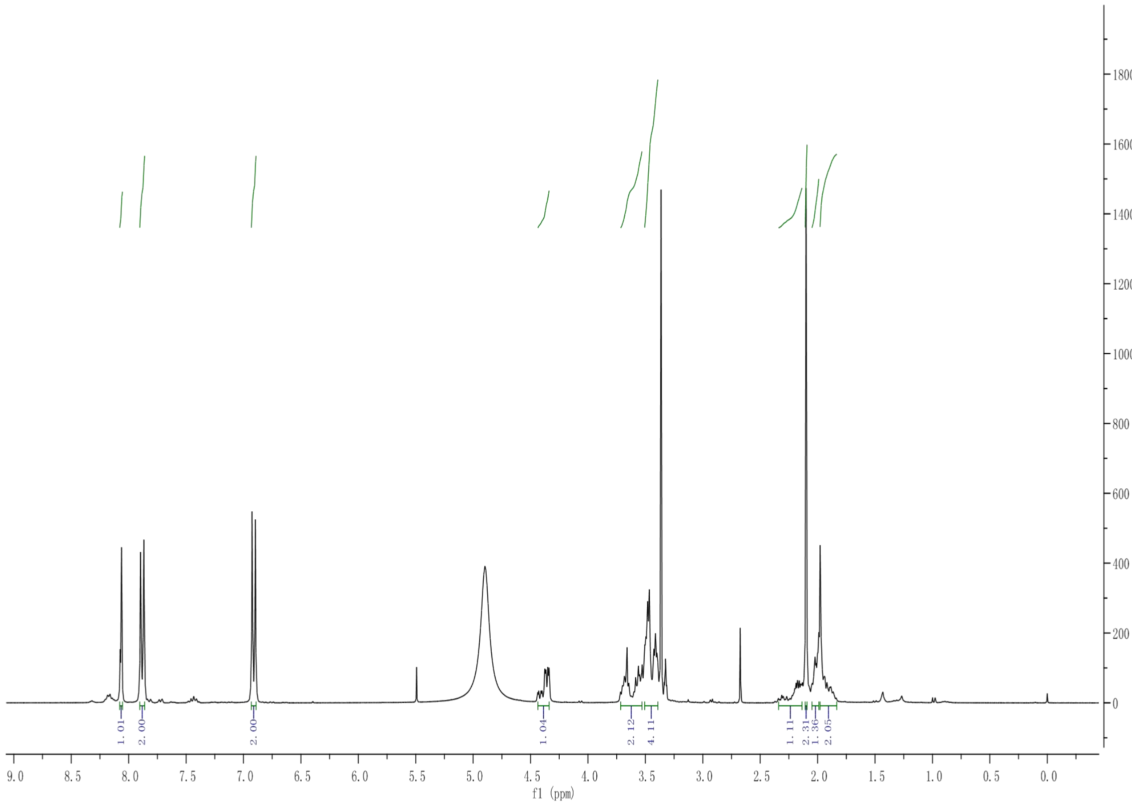

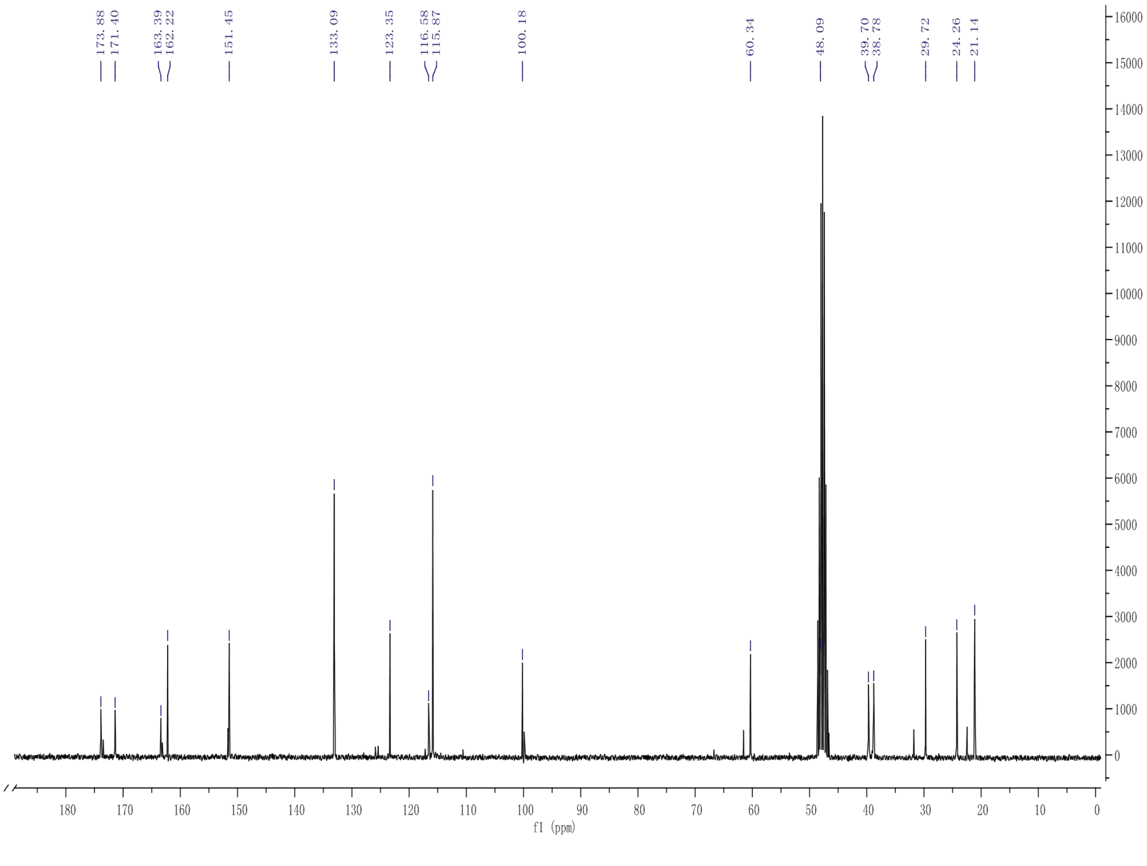

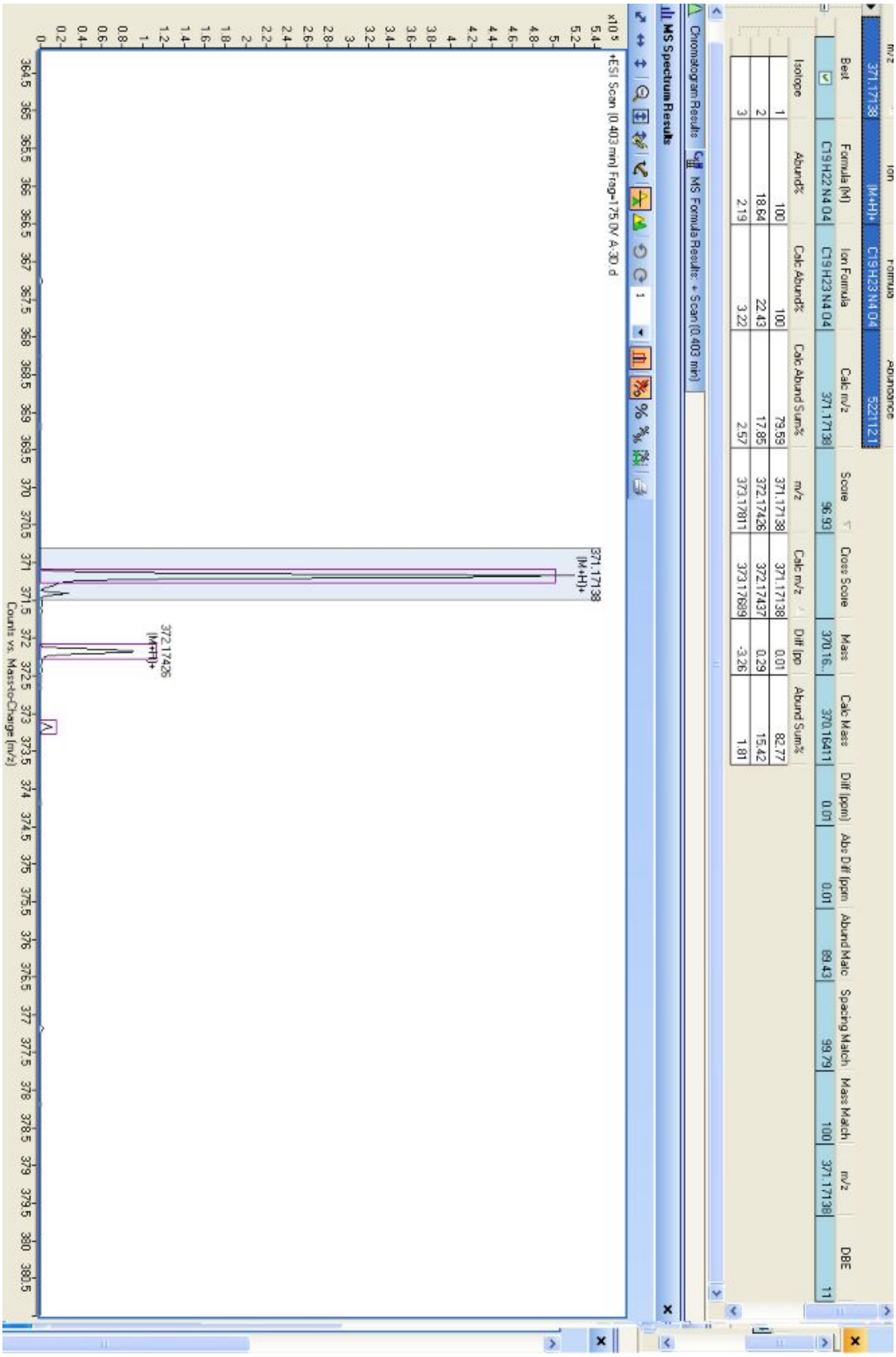
MS Spectrum Results
+ESI Scan (0.403 min) Frag=175.0V A-30.d
371.17138
(M+H)+
372.17426
(M+H)+
Counts vs. Mass-to-Charge (m/z)
C19 H22 N4 O4
C19 H23 N4 O4

5c (^{1}H NMR, ^{13}C NMR, HRMS)

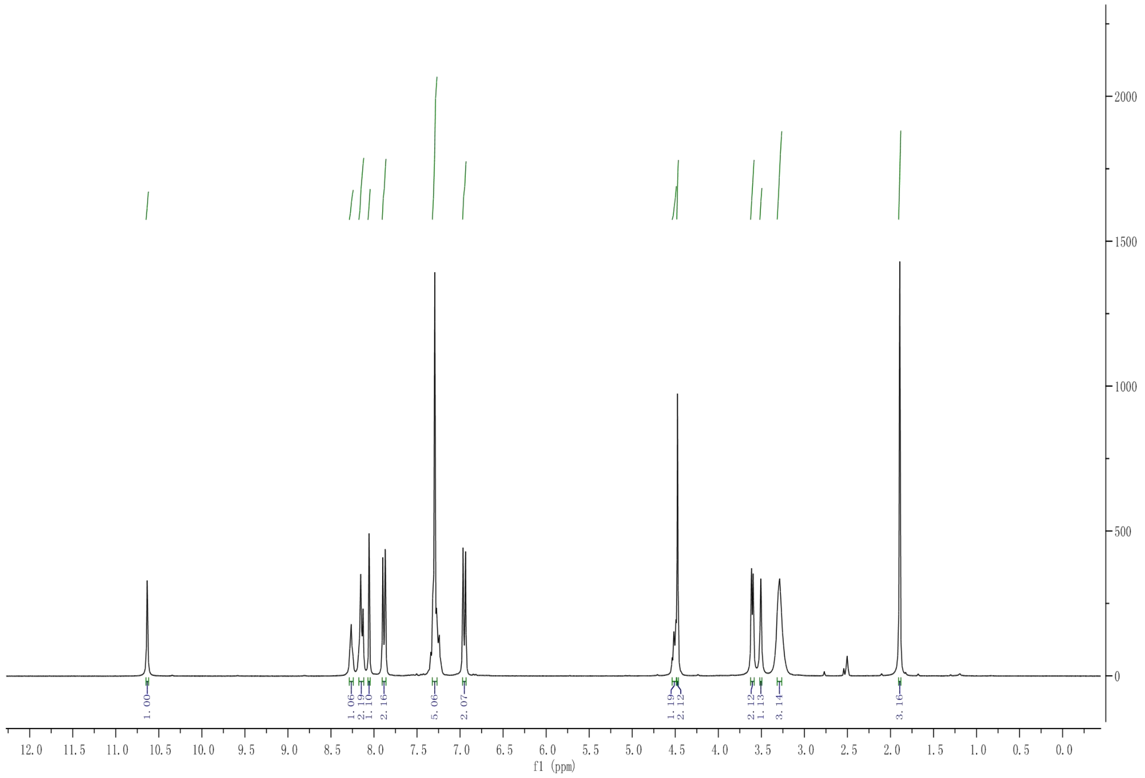

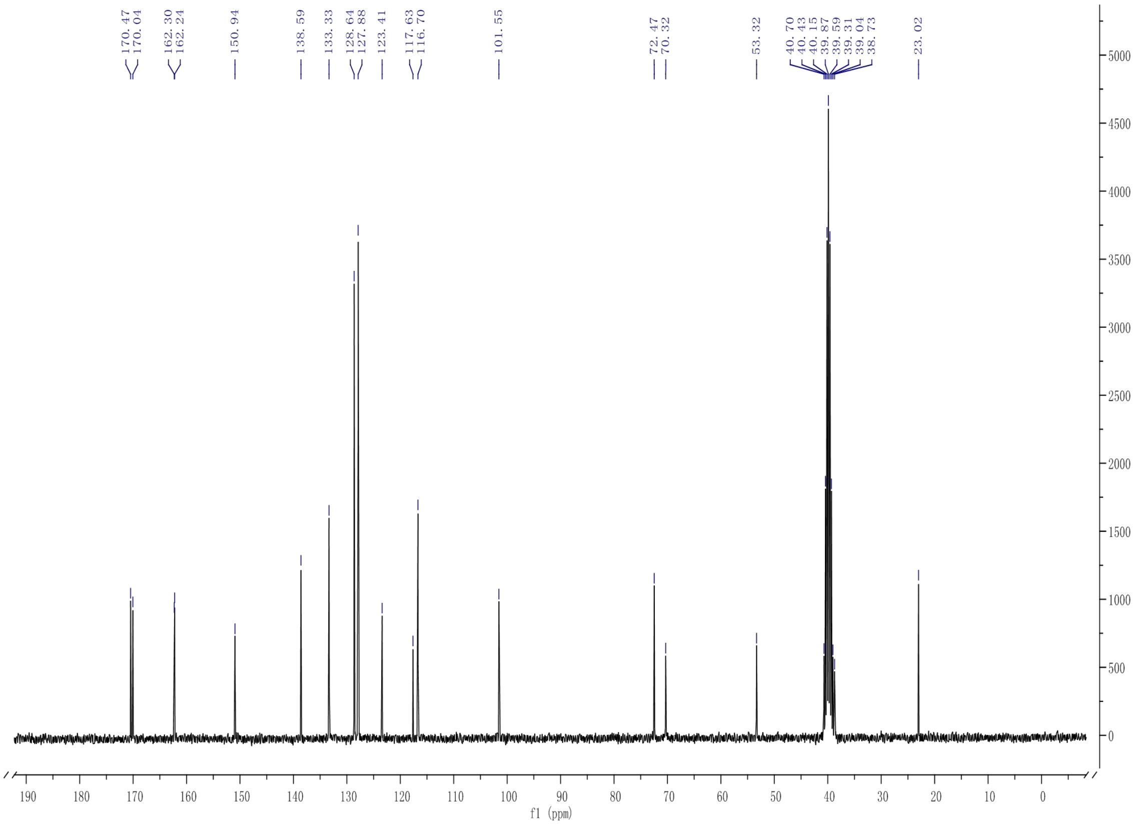

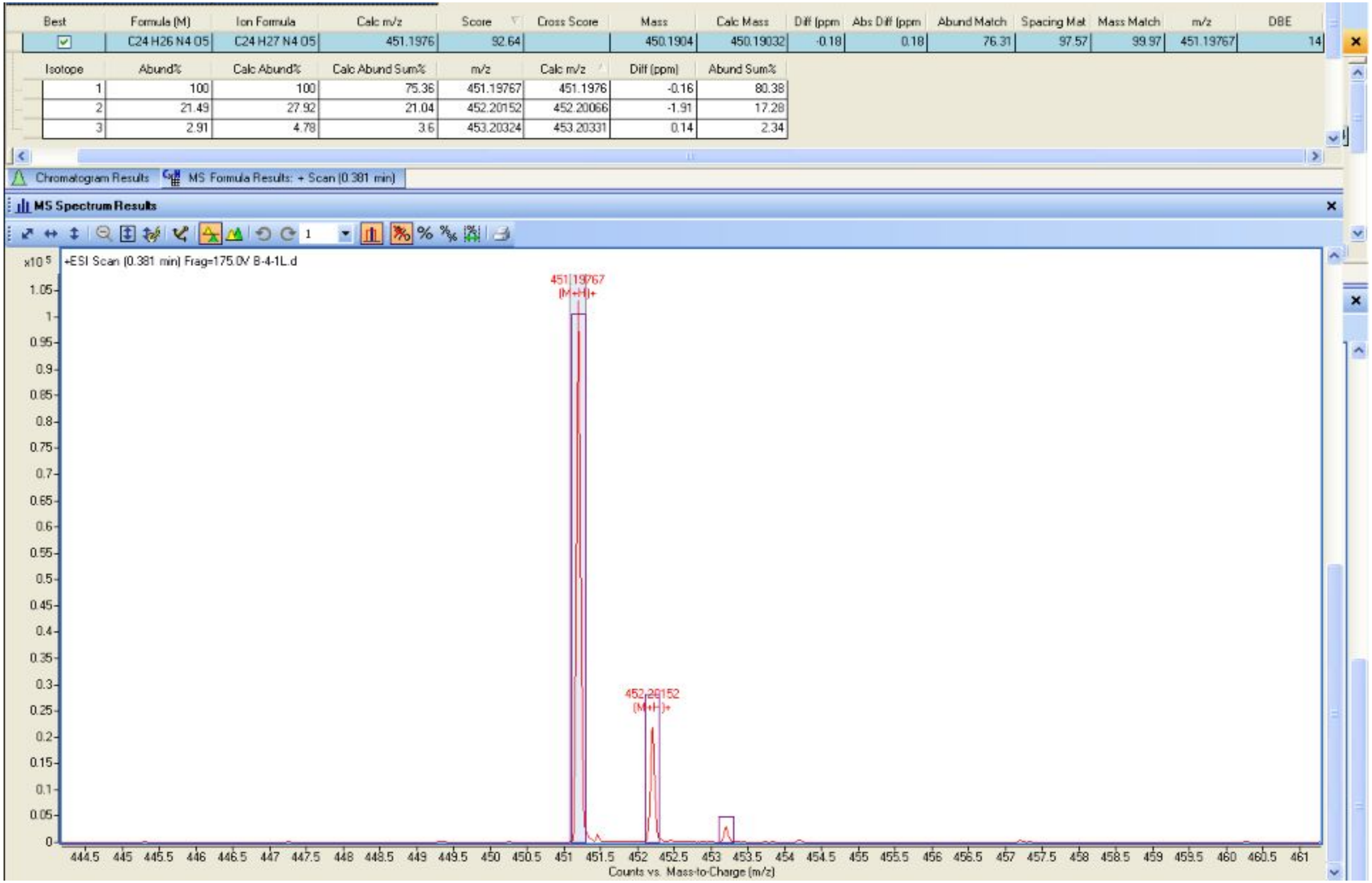

Best	Formula (M)	Ion Formula	Calc m/z	Score	Cross Score	Mass	Calc Mass	Diff (ppm	Abs Diff (ppm	Abund Match	Spacing Mat	Mass Match	m/z	DBE
☑	C24 H26 N4 O5	C24 H27 N4 O5	451.1976	92.64		450.1904	450.19032	-0.18	0.18	76.31	97.57	99.97	451.19767	14

Isotope	Abund%	Calc Abund%	Calc Abund Sum%	m/z	Calc m/z	Diff (ppm)	Abund Sum%
1	100	100	75.36	451.19767	451.1976	-0.16	80.38
2	21.49	27.92	21.04	452.20152	452.20066	-1.91	17.28
3	2.91	4.78	3.6	453.20324	453.20331	0.14	2.34

5d (^{1}H NMR, ^{13}C NMR, HRMS)

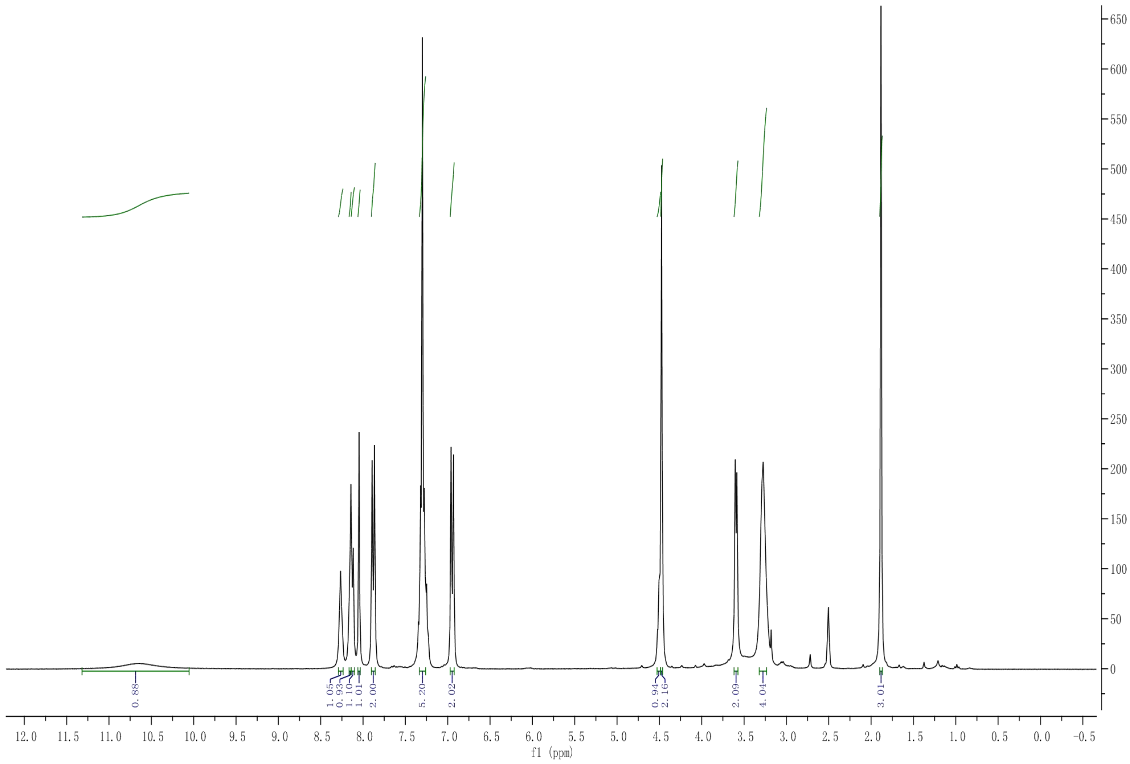

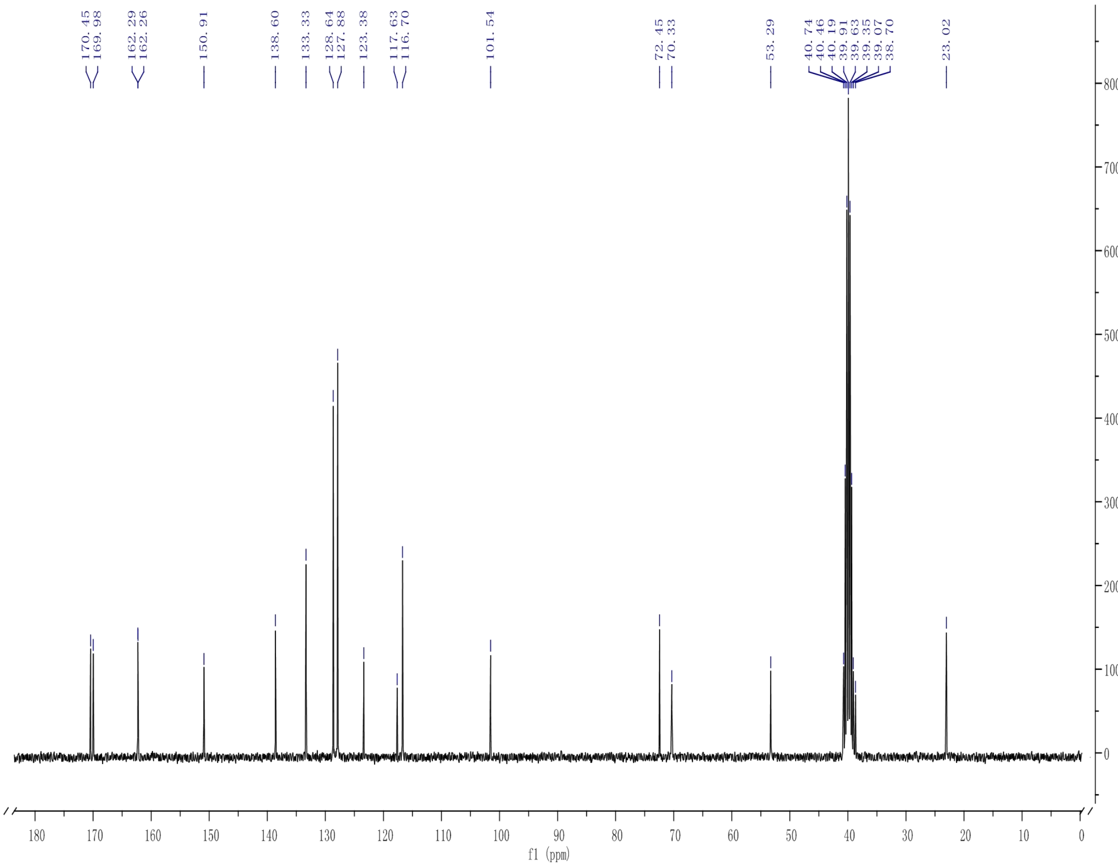

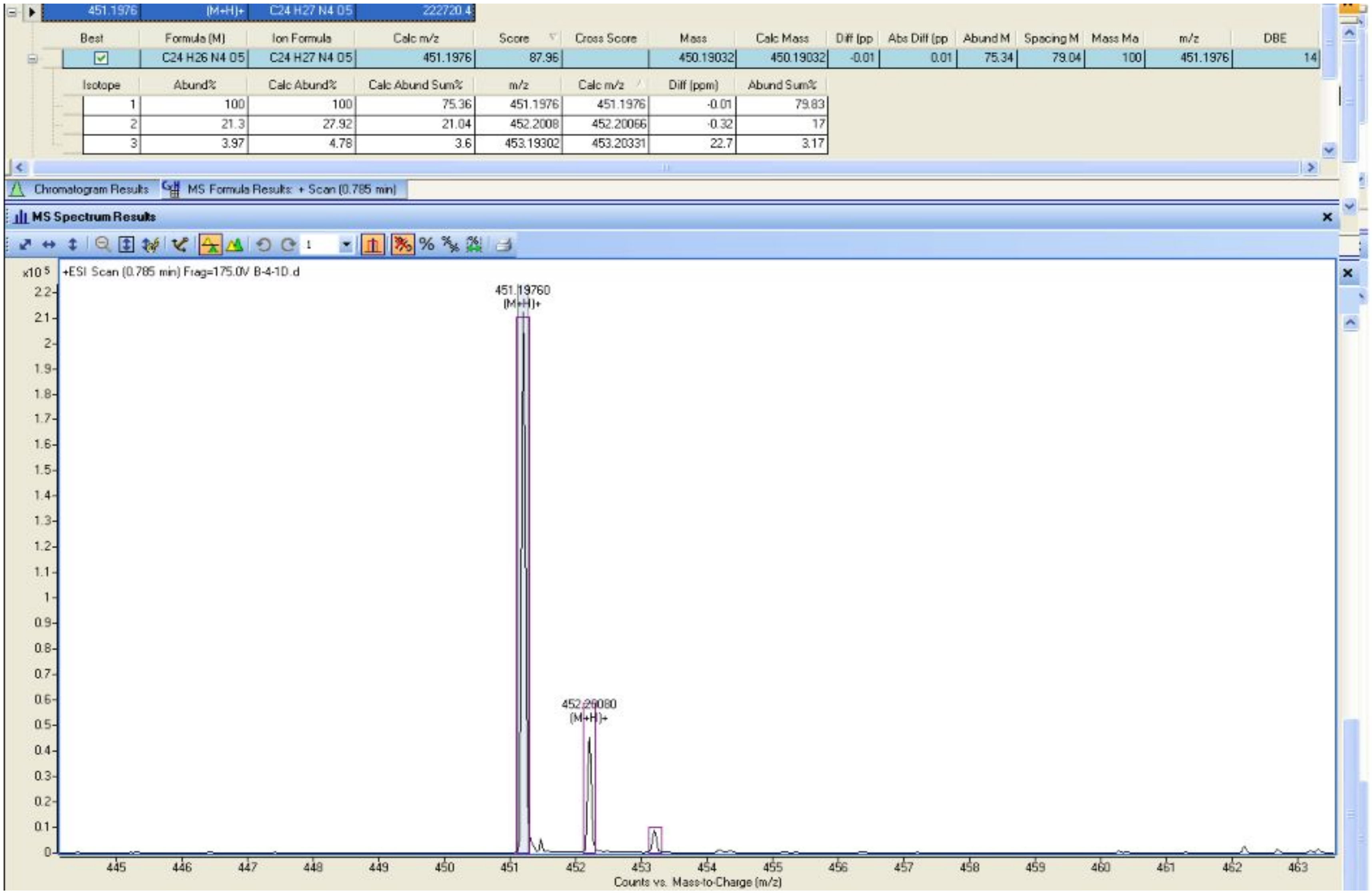

451.1976	(M+H)+	C24 H27 N4 O5	222720.4

Best	Formula (M)	Ion Formula	Calc m/z	Score	Cross Score	Mass	Calc Mass	Diff (pp	Abs Diff (pp	Abund M	Spacing M	Mass Ma	m/z	DBE
☑	C24 H26 N4 O5	C24 H27 N4 O5	451.1976	87.96		450.19032	450.19032	-0.01	0.01	75.34	79.04	100	451.1976	14

Isotope	Abund%	Calc Abund%	Calc Abund Sum%	m/z	Calc m/z	Diff (ppm)	Abund Sum%
1	100	100	75.36	451.1976	451.1976	-0.01	79.83
2	21.3	27.92	21.04	452.2008	452.20066	-0.32	17
3	3.97	4.78	3.6	453.19302	453.20331	22.7	3.17

5e (^{1}H NMR, ^{13}C NMR, HRMS)

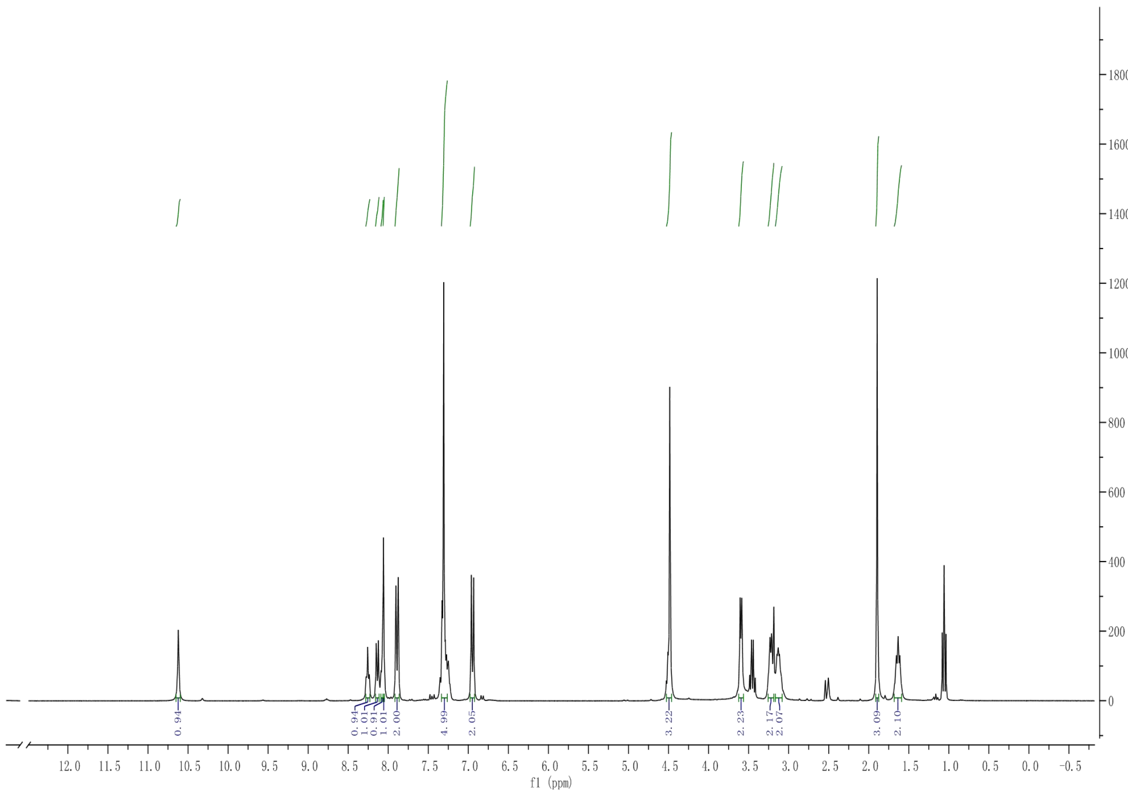

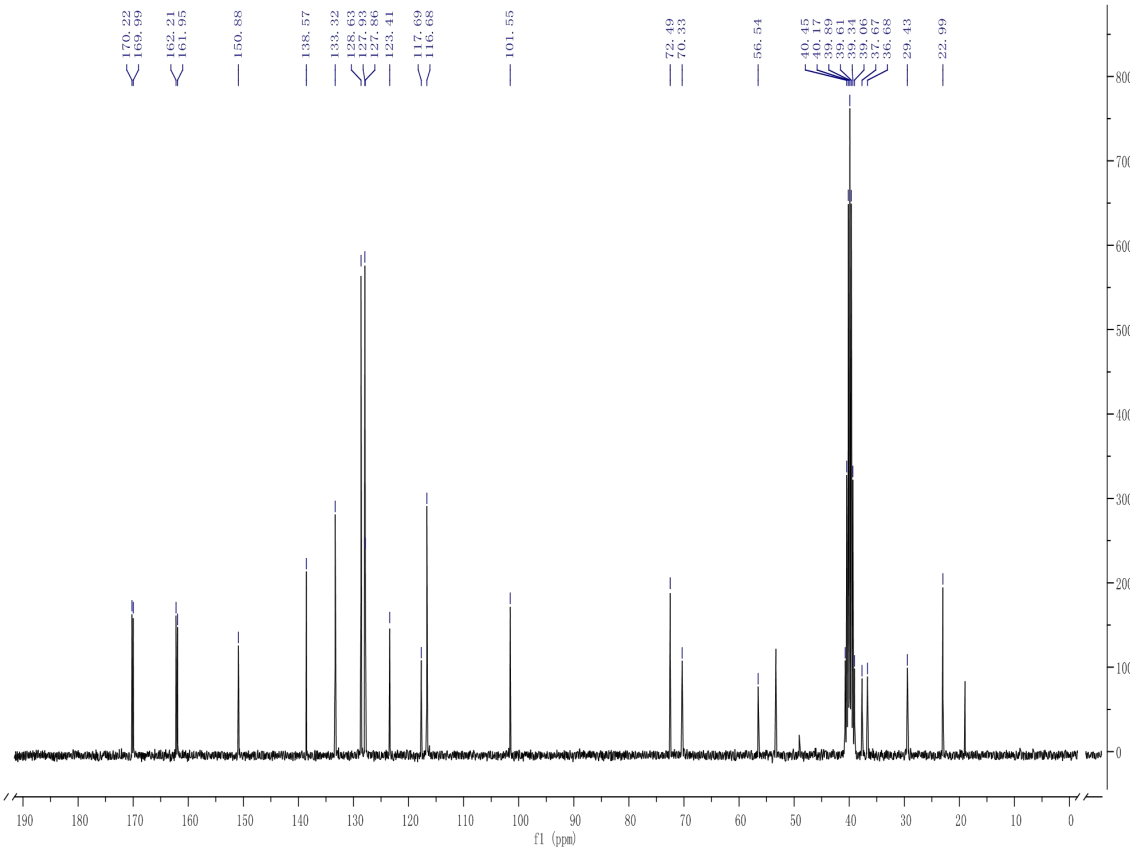

m/z	Ion	Formula	Abundance
465.21327	(M+H)+	C25 H29 N4 O5	976694

Best	Formula (M)	Ion Formula	Calc m/z	Score	Cross Score	Mass	Calc Mass	Diff (ppm)	Abs Diff (pp	Abund Match	Spacing Matc	Mass Match	m/z	DBE
☑	C25 H28 N4 O5	C25 H29 N4 O5	465.21325	98.53		464.20597	464.20597	0	0	97.36	96.99	100	465.21327	14

Isotope	Abund%	Calc Abund%	Calc Abund Su	m/z	Calc m/z	Diff (ppm)	Abund Sum%
1	100	100	74.56	465.21327	465.21325	-0.05	76
2	28.06	29.02	21.64	466.21545	466.21632	1.88	21.33
3	3.52	5.09	3.8	467.21874	467.219	0.55	2.67

Chromatogram Results | MS Formula Results: + Scan (0.586 min)

MS Spectrum Results

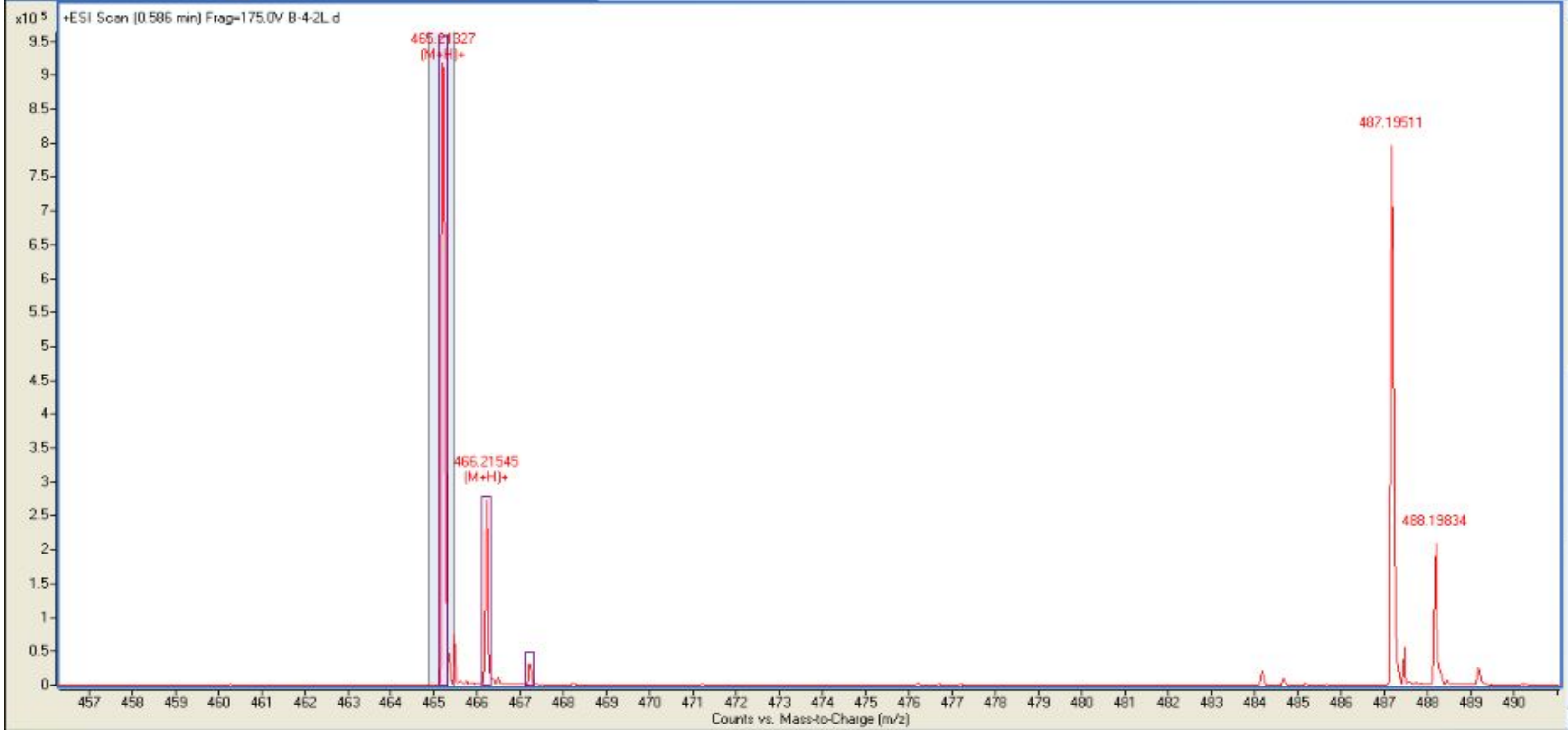

5f (^{1}H NMR, ^{13}C NMR, HRMS)

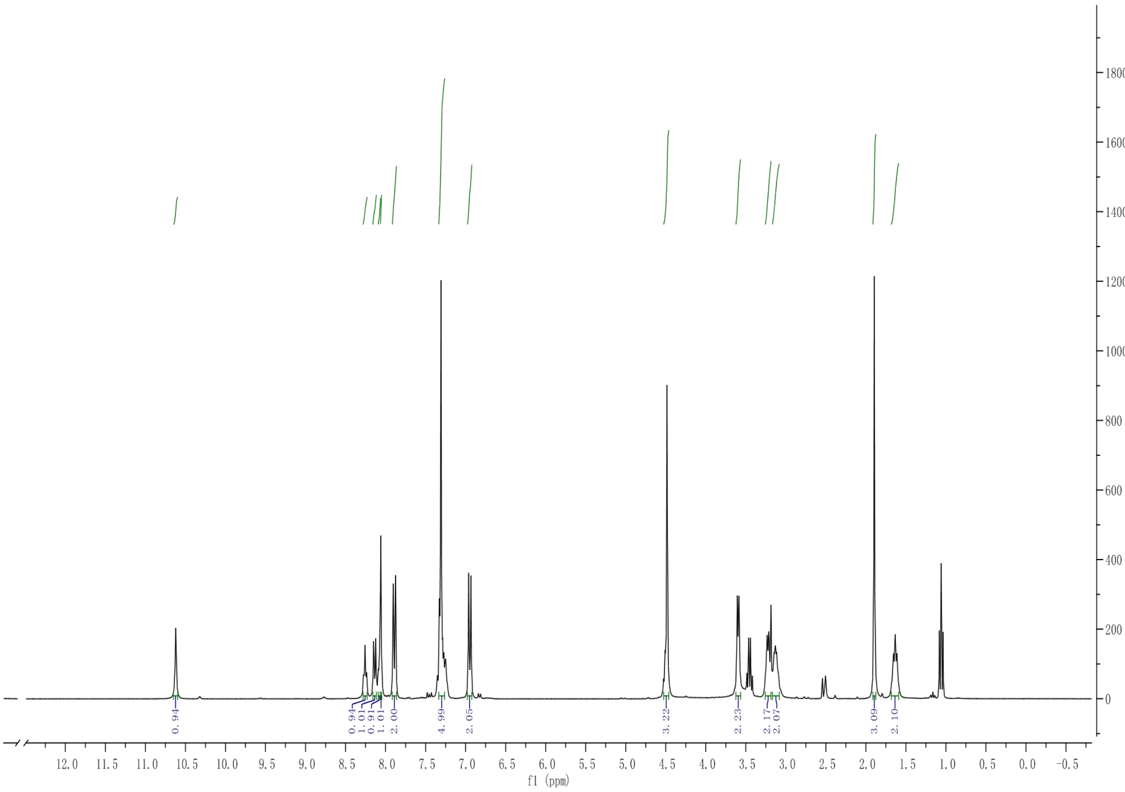

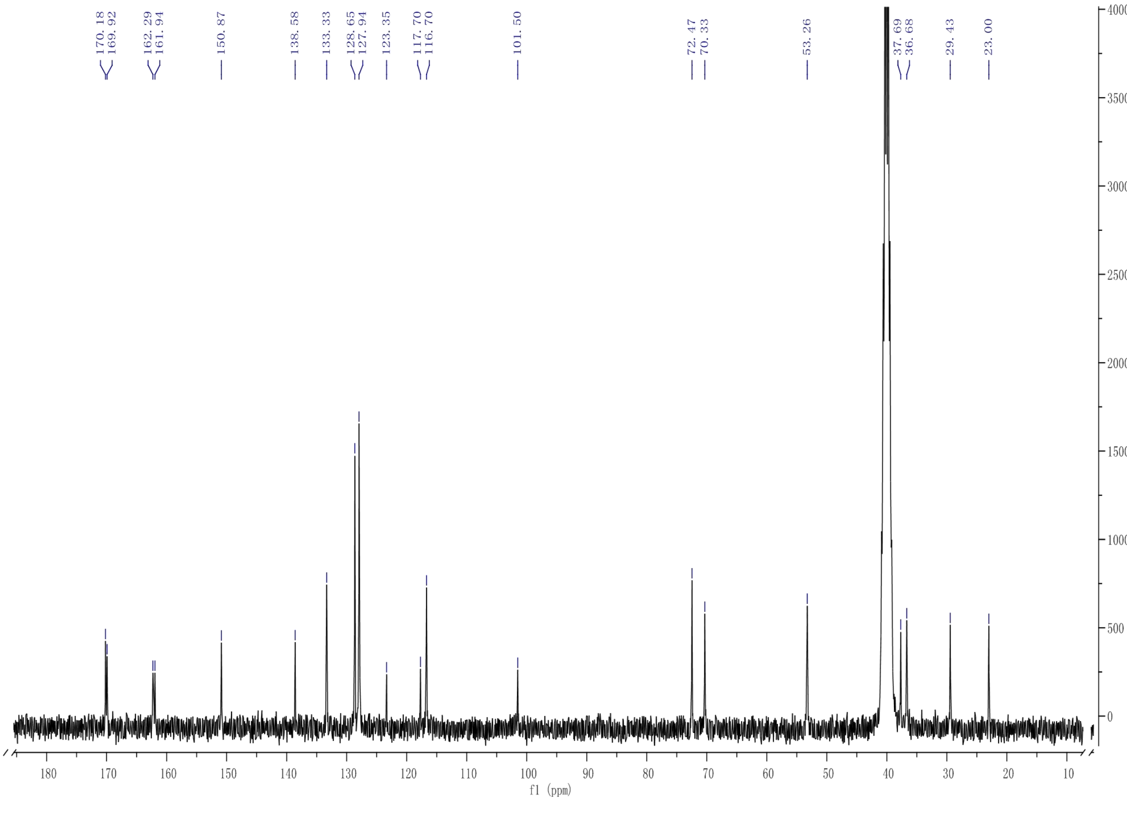

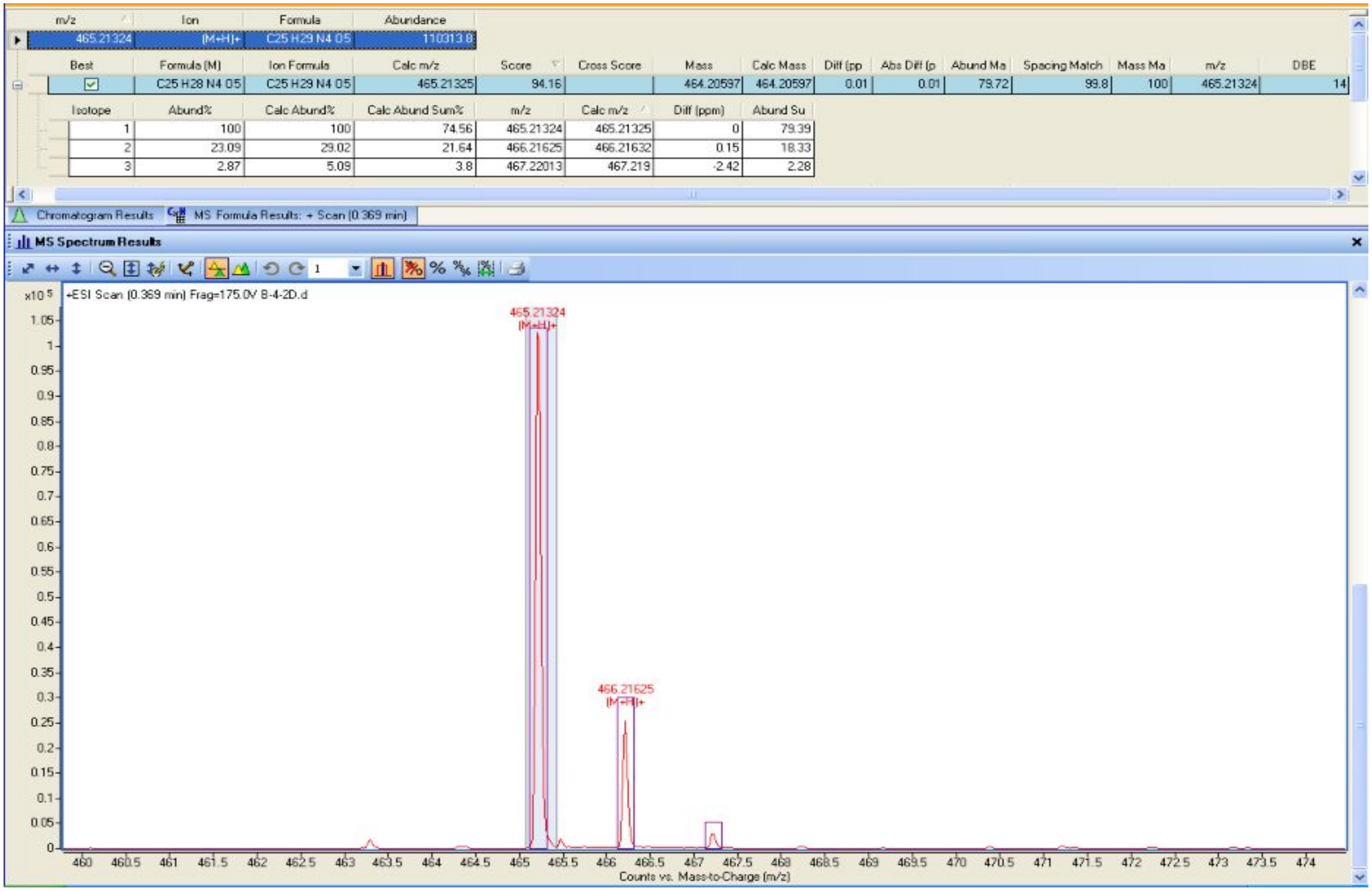
m/z | Ion | Formula | Abundance
465.21324 | [M+H]+ | C25 H29 N4 O5 | 110313.8
Best | Formula (M) | Ion Formula | Calc m/z | Score | Cross Score | Mass | Calc Mass | Diff (pp | Abs Diff (p | Abund Ma | Spacing Match | Mass Ma | m/z | DBE
C25 H28 N4 O5 | C25 H29 N4 O5 | 465.21325 | 94.16 | 464.20597 | 464.20597 | 0.01 | 0.01 | 79.72 | 99.8 | 100 | 465.21324 | 14
Isotope | Abund% | Calc Abund% | Calc Abund Sum% | m/z | Calc m/z | Diff (ppm) | Abund Su
1 | 100 | 100 | 74.56 | 465.21324 | 465.21325 | 0 | 79.39
2 | 23.09 | 29.02 | 21.64 | 466.21625 | 466.21632 | 0.15 | 18.33
3 | 2.87 | 5.09 | 3.8 | 467.22013 | 467.219 | -2.42 | 2.28
Chromatogram Results
MS Formula Results: + Scan (0.369 min)
MS Spectrum Results
x10 5
+ESI Scan (0.369 min) Frag=175.0V B-4-2D.d
465.21324
[M+H]+
466.21625
[M+H]+
Counts vs. Mass-to-Charge (m/z)

5g (^{1}H NMR, ^{13}C NMR, HRMS)

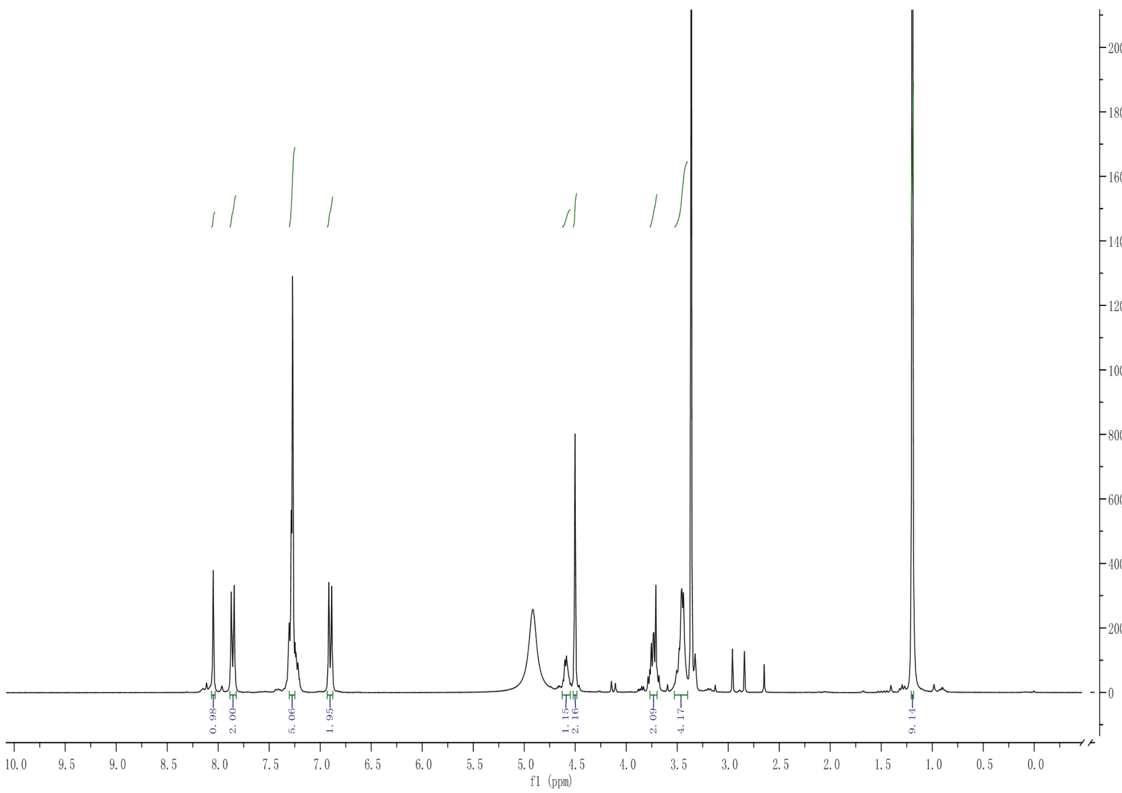

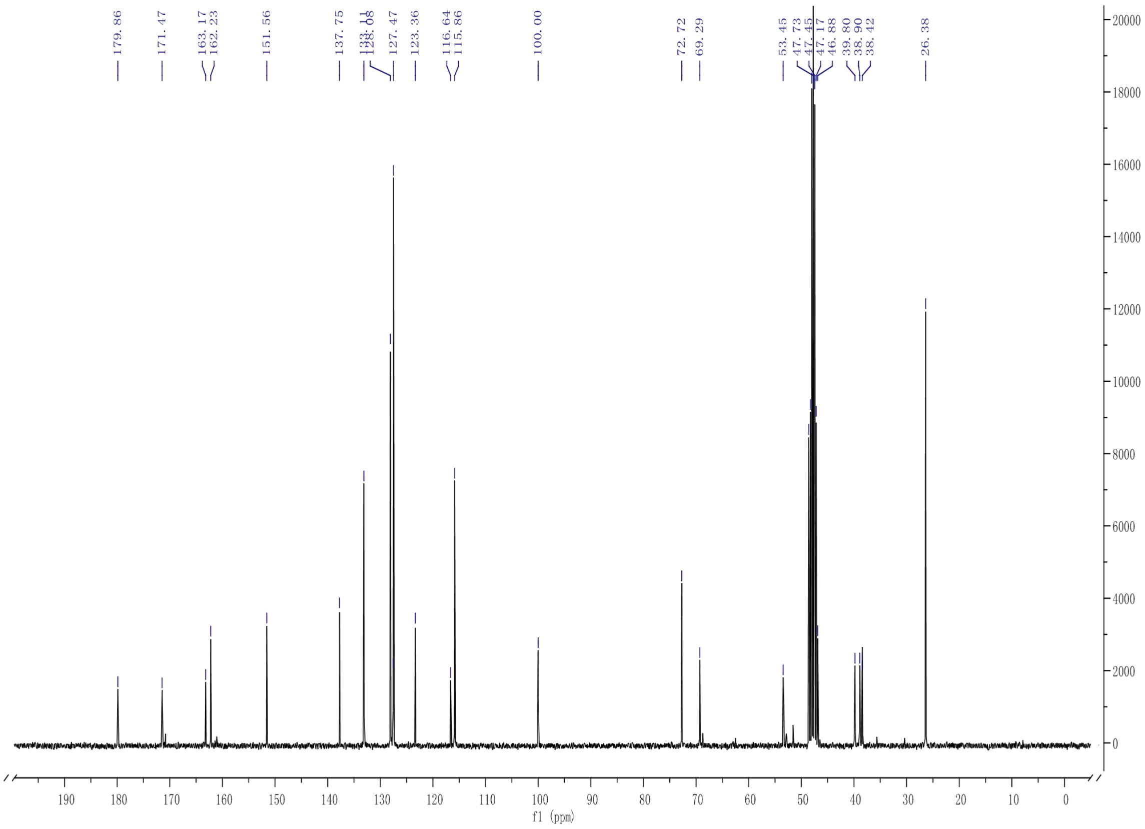

m/z	Ion	Formula	Abundance
493.24452	(M+H)+	C27 H33 N4 O5	55089.5

Best	Formula (M)	Ion Formula	Calc m/z	Score	Cross Score	Mass	Calc Mass	Diff (ppm)	Abs Diff (pp	Abund Match	Spacing M	Mass Mat	m/z	DBE
☑	C27 H32 N4 O5	C27 H33 N4 O5	493.24455	94.62		492.23724	492.23727	0.06	0.06	81.61	99.5	100	493.24452	14

Isotope	Abund%	Calc Abund%	Calc Abund Sum%	m/z	Calc m/z	Diff (ppm)	Abund Sum%
1	100	100	73	493.24452	493.24455	0.06	77.37
2	25.22	31.23	22.8	494.24751	494.24765	0.28	19.51
3	4.03	5.74	4.19	495.25169	495.25039	-2.63	3.12

Chromatogram Results | MS Formula Results: + Scan (1.360 min)

MS Spectrum Results

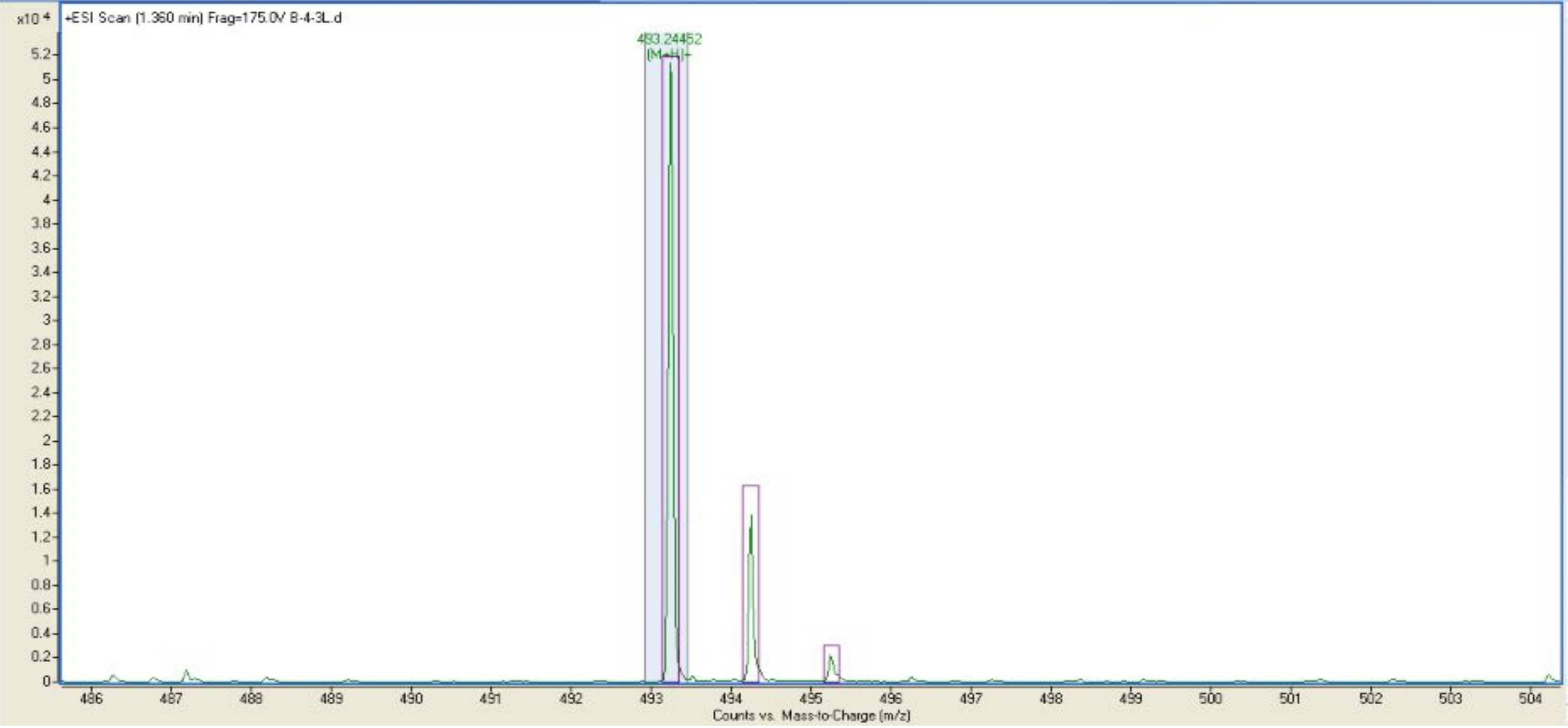

5h (^{1}H NMR, ^{13}C NMR, HRMS)

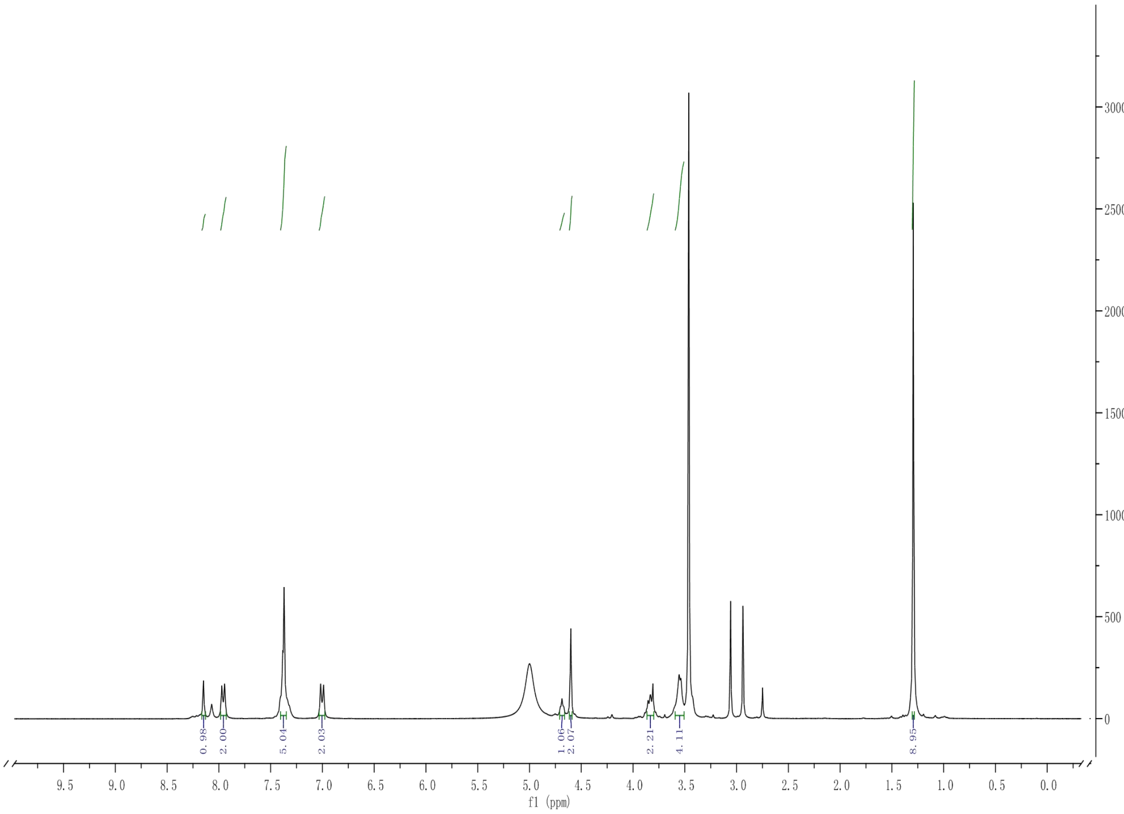

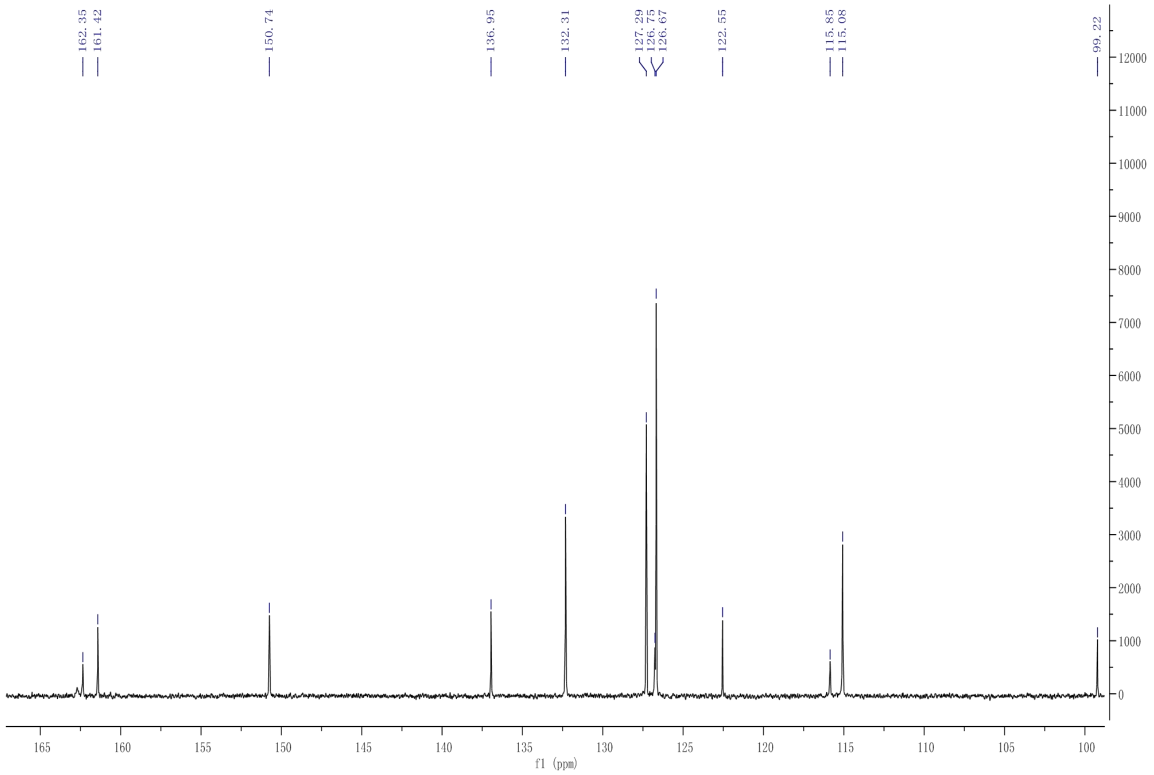

m/z	Ion	Formula	Abundance
493.24454	(M+H)+	C27 H33 N4 O5	758597.3

Best	Formula (M)	Ion Formula	Calc m/z	Score	Cross Score	Mass	Calc Mass	Diff (ppm)	Abs Diff (ppm)	Abund Match	Spacing Match	Mass Match
☑	C27 H32 N4 O5	C27 H33 N4 O5	493.24455	98.72		492.23725	492.23727	0.03	0.03	96.33	99.02	100

Isotope	Abund%	Calc Abund%	Calc Abund Sum%	m/z	Calc m/z	Diff (ppm)	Abund Sum%
1	100	100	73	493.24454	493.24455	0	74.86
2	29.69	31.23	22.8	494.24714	494.24765	1.03	22.23
3	3.89	5.74	4.19	495.24986	495.25039	1.06	2.91

Chromatogram Results | MS Formula Results: + Scan (0.617 min)

MS Spectrum Results

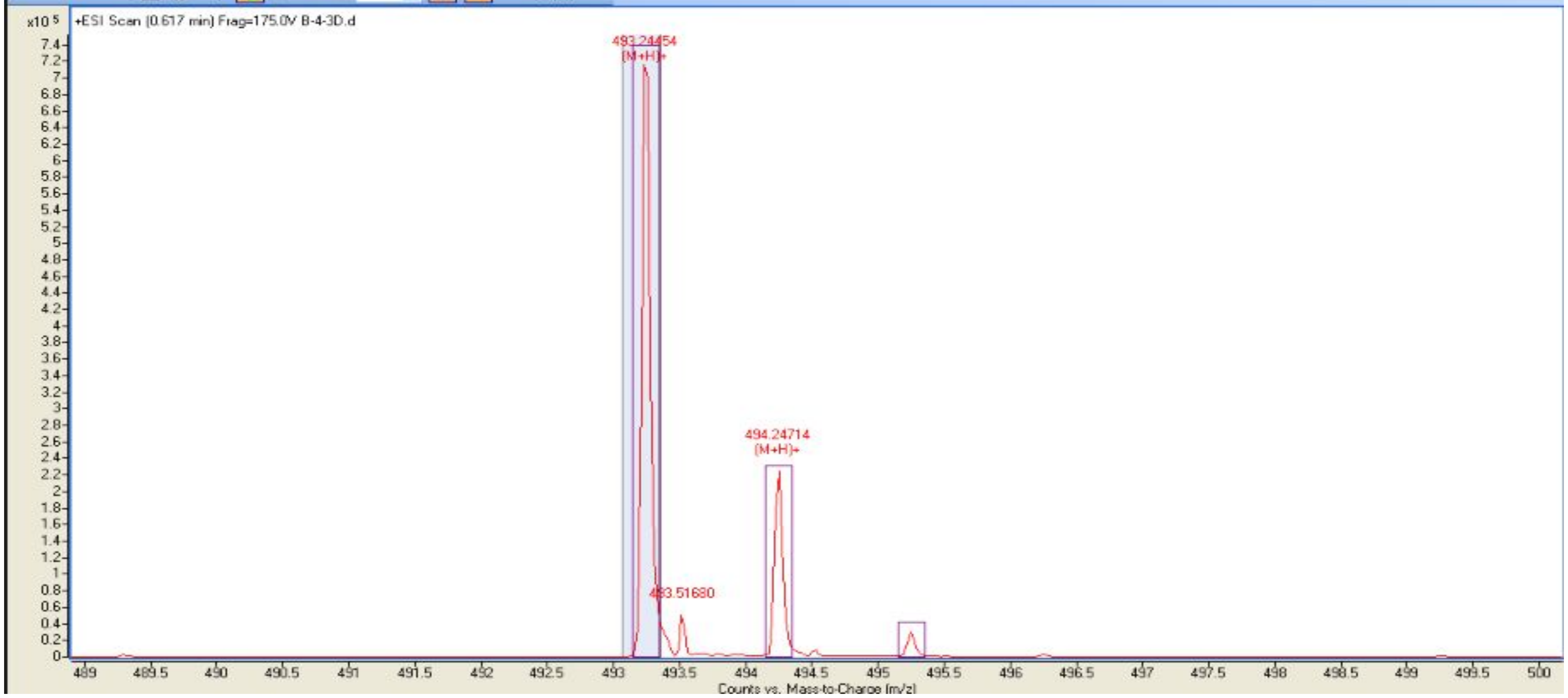

5i (^{1}H NMR, ^{13}C NMR, HRMS)

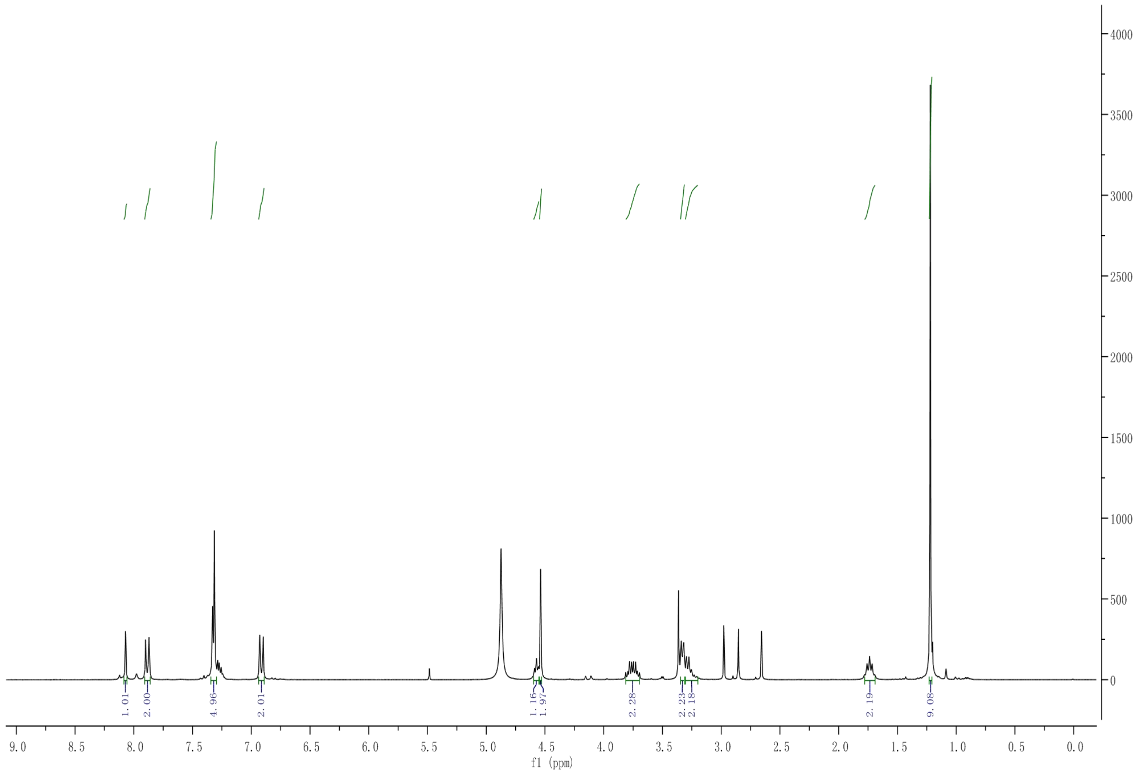

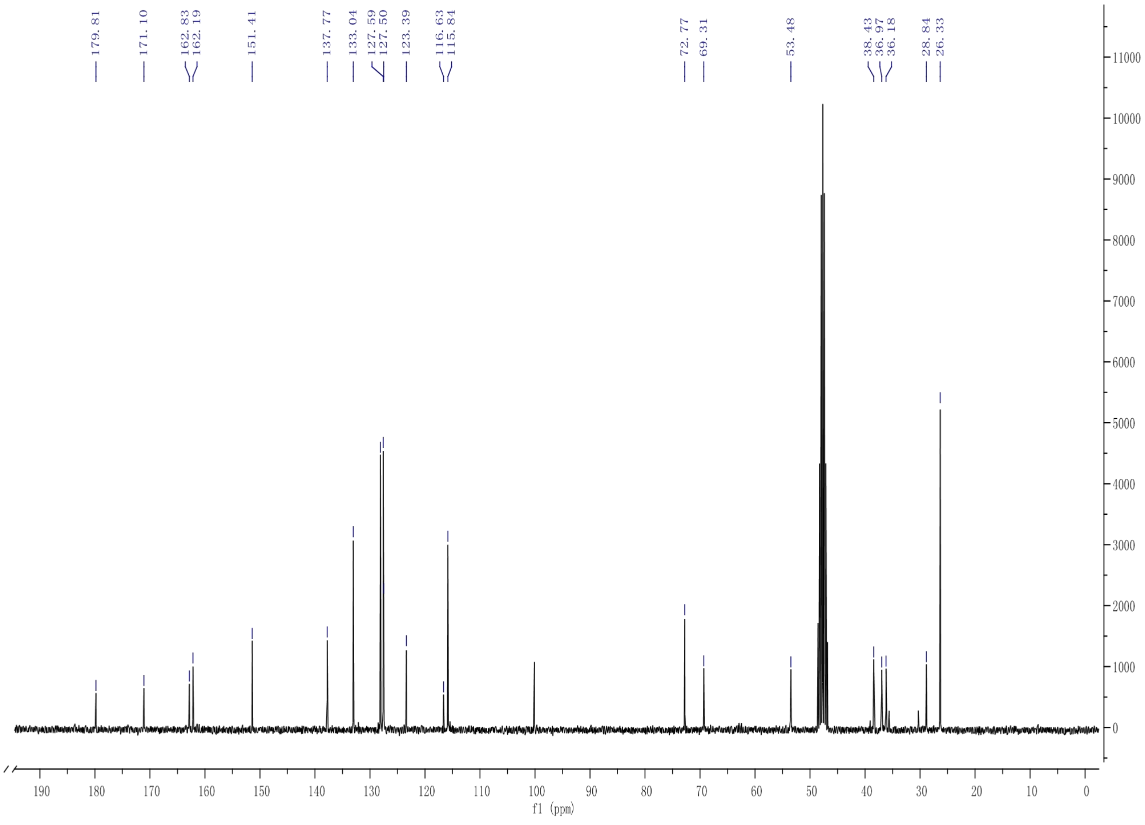

m/z	Ion	Formula	Abundance
507.26022	[M+H]+	C28 H35 N4 O5	220562.6

Best	Formula (M)	Ion Formula	Calc m/z	Score	Cross Score	Mass	Calc Mass	Diff (ppm)	Abs Diff (ppm)	Abund Match	Spacing Match	Mass Match	m/z
☑	C28 H34 N4 O5	C28 H35 N4 O5	507.2602	95.11		506.25295	506.25292	-0.06	0.06	83.15	99.7	100	507.2602

Isotope	Abund%	Calc Abund%	Calc Abund Sum%	m/z	Calc m/z	Diff (ppm)	Abund Sum%
1	100	100	72.24	507.26022	507.2602	-0.05	76.68
2	26.63	32.34	23.36	508.2636	508.26331	-0.58	20.42
3	3.78	6.09	4.4	509.26652	509.26608	-0.88	2.9

Chromatogram Results | MS Formula Results: + Scan (0.395 min)

MS Spectrum Results

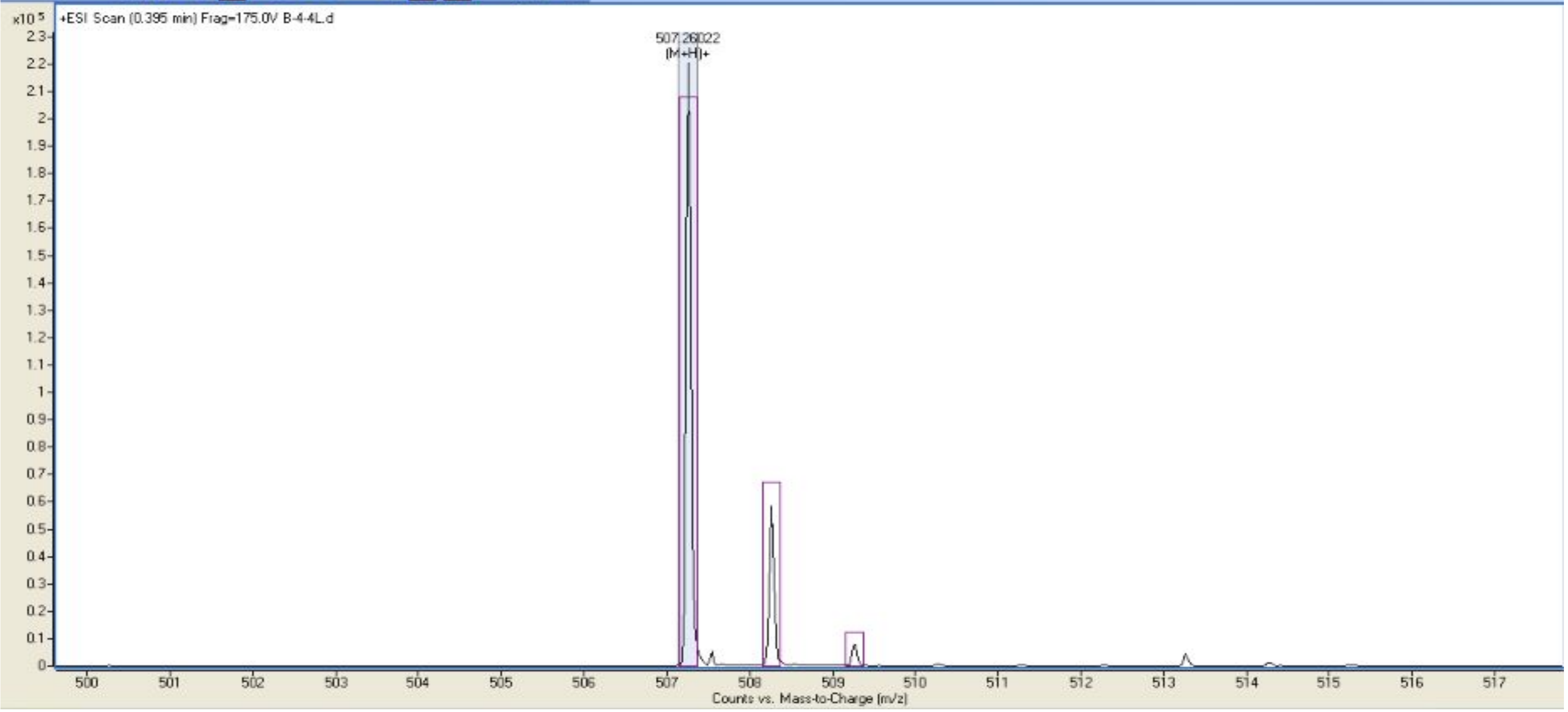

5j (^{1}H NMR, ^{13}C NMR, HRMS)

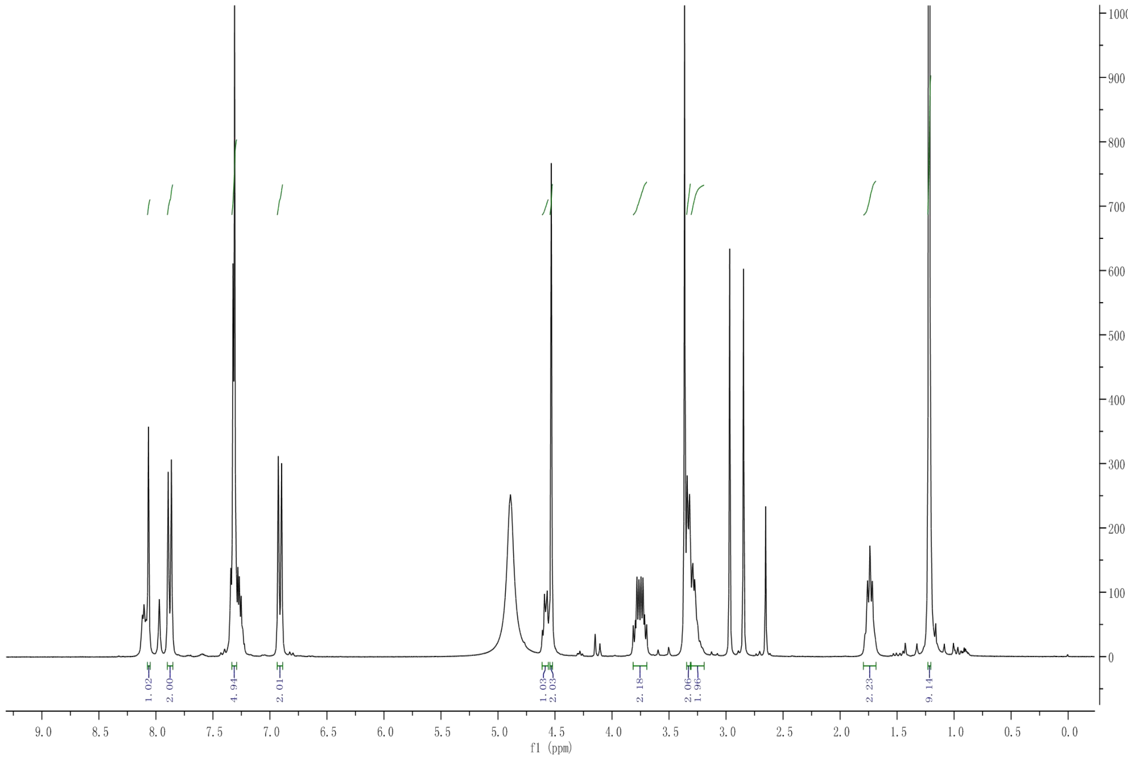

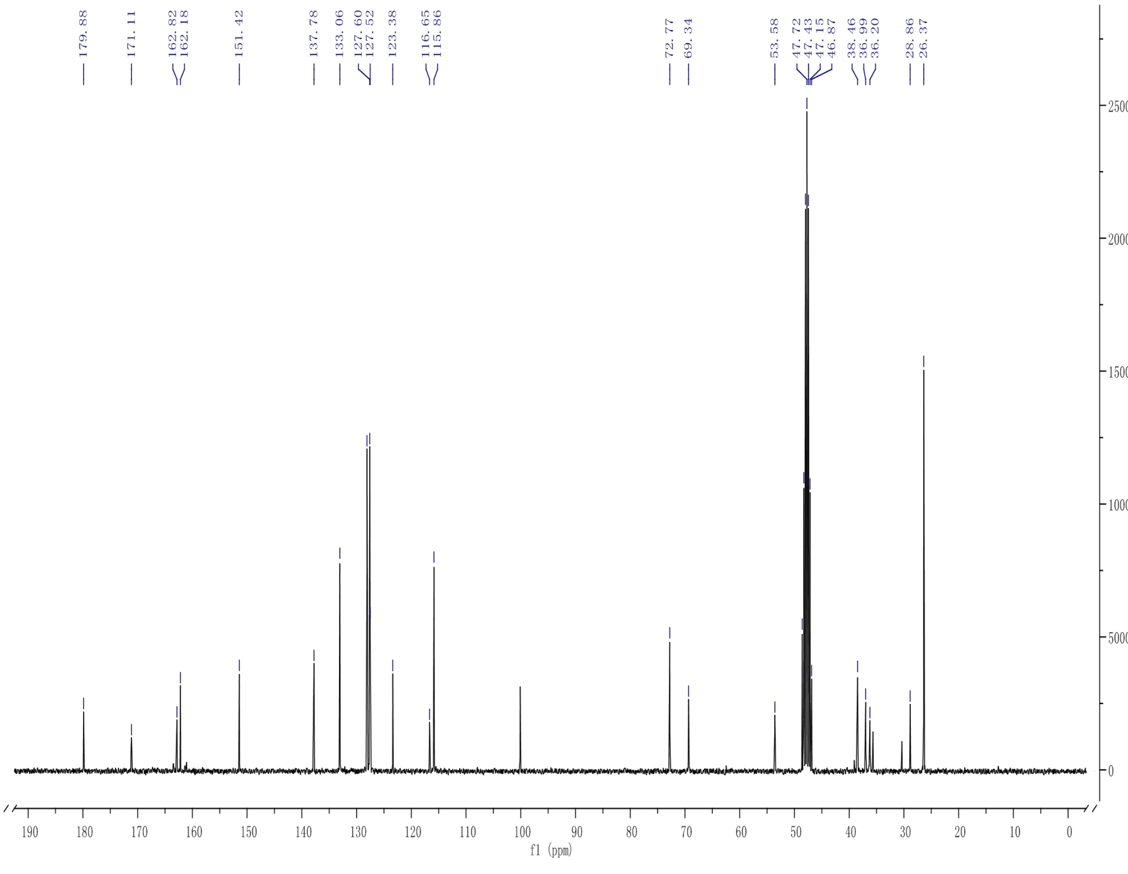

m/z	Ion	Formula	Abundance
507.26055	(M+H)+	C28 H35 N4 O5	40224.8

Best	Formula (M)	Ion Formula	Calc m/z	Score	Cross Score	Mass	Calc Mass	Diff (ppm)	Abs Diff (ppm)	Abund Match	Spacing Match	Mass Match	m/z
☑	C28 H34 N4 O5	C28 H35 N4 O5	507.2602	92.01		506.25329	506.25292	-0.72	0.72	79.83	91.77	99.43	507.26055

Isotope	Abund%	Calc Abund%	Calc Abund Sum%	m/z	Calc m/z	Diff (ppm)	Abund Sum%
1	100	100	71.8	507.26055	507.2602	-0.7	74.91
2	26.25	32.34	23.22	508.26431	508.26331	-1.98	19.67
3	5.93	6.09	4.37	509.27012	509.26608	-7.94	4.45
4	1.3	0.84	0.61	510.27483	510.26873	-11.94	0.98

Chromatogram Results | MS Formula Results: + Scan (1.252 min)

MS Spectrum Results

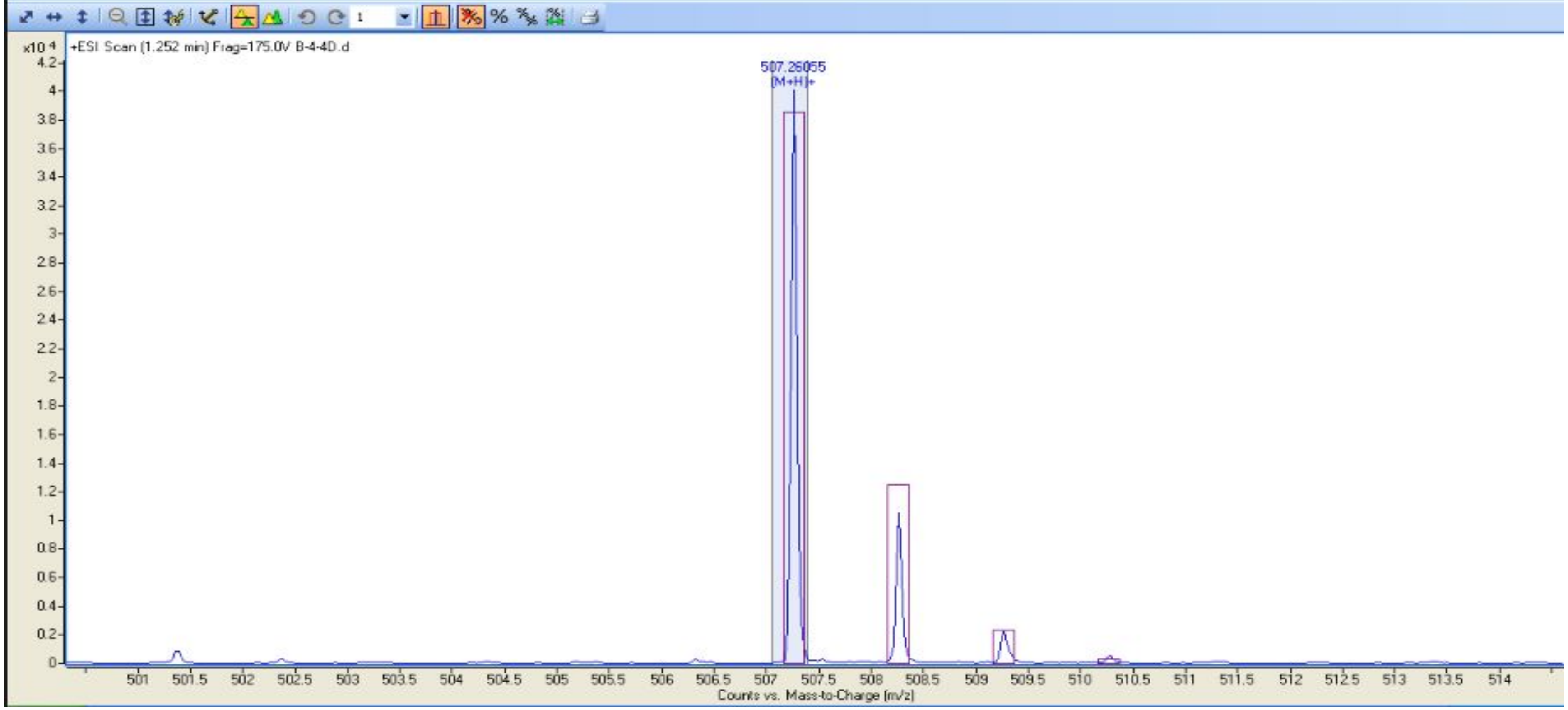

II. Molecular docking

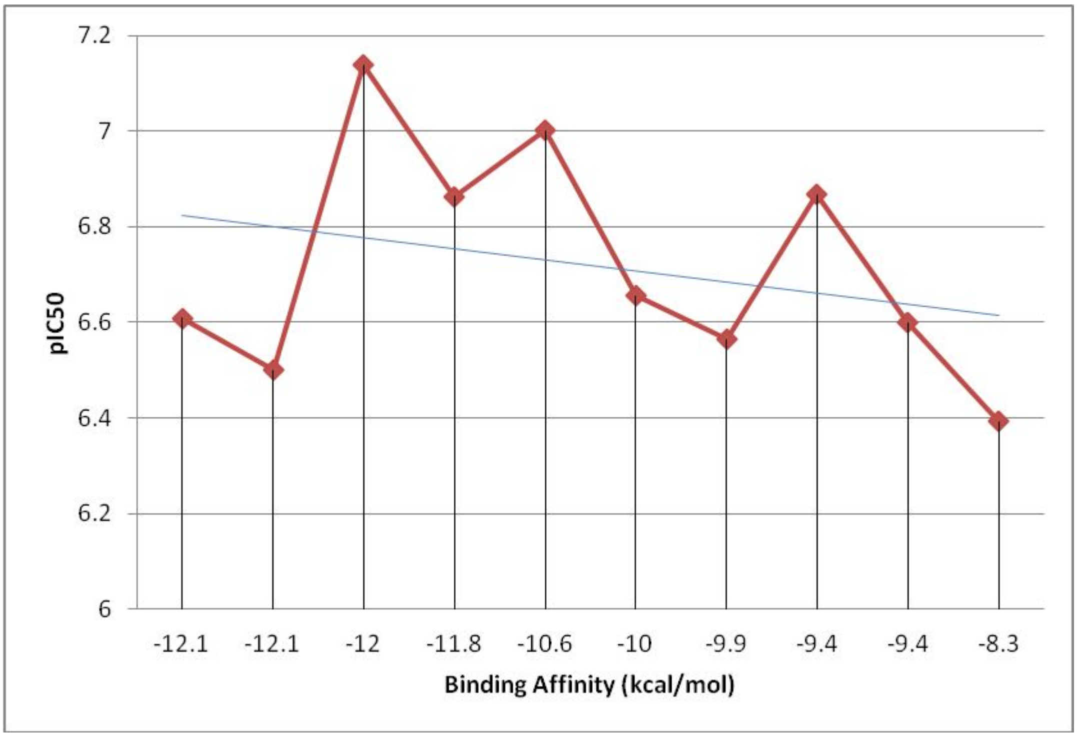

Fig. S4. The plot of binding affinity (kcal/mol) and pIC_{50} (the blue line shows the linear correlation between the binding affinity and pIC50 value).

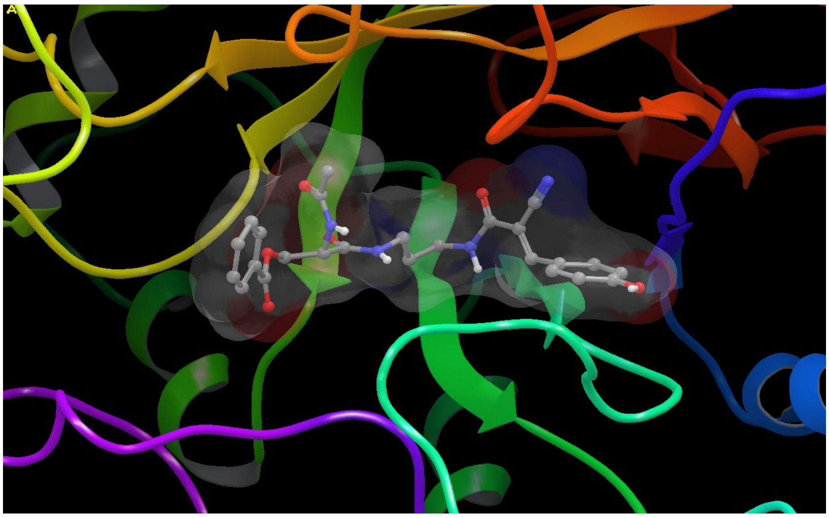

Fig. S5. **5e** poses at the binding sites.

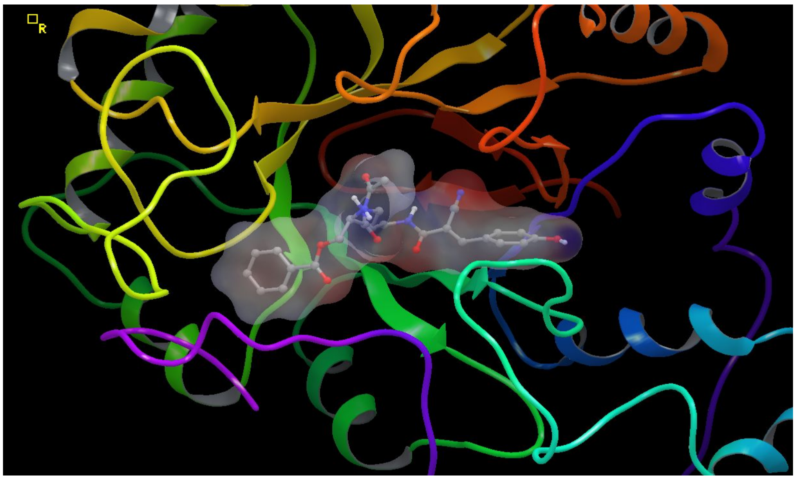

Fig. S6. **5f** poses at the binding sites.

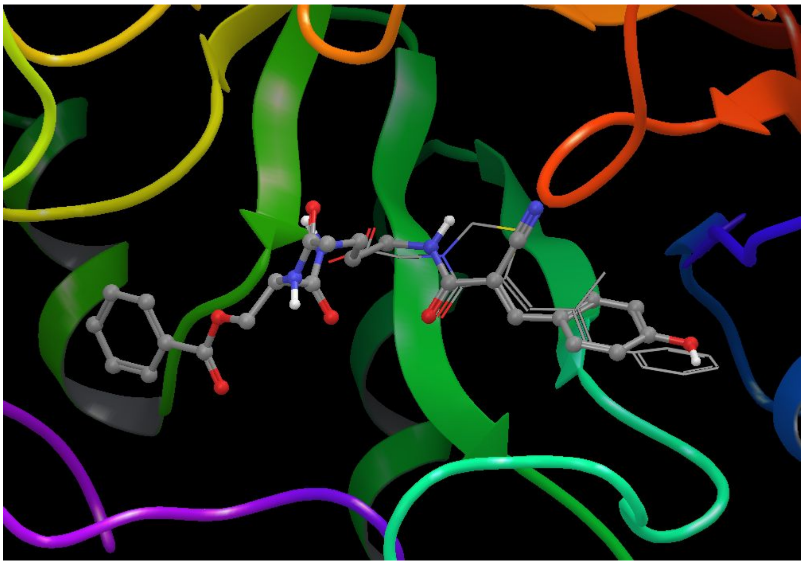

Fig. S7. **5f** superimpositions with Epalrest at the binding sites.

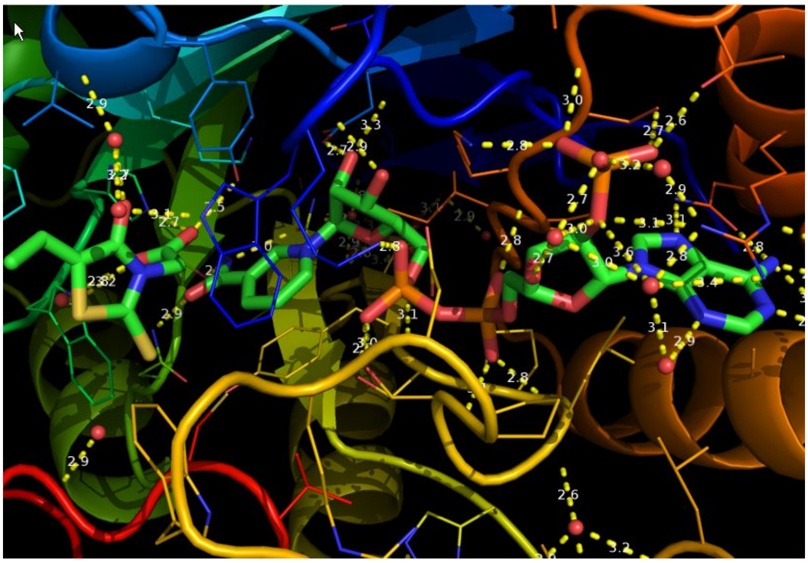

Fig. S8. Aldose reductase with Epalrestat which was catalyzed in the active site
